# Single Sample Expression-Anchored Mechanisms Predict Survival in Head and Neck Cancer

**DOI:** 10.1371/journal.pcbi.1002350

**Published:** 2012-01-26

**Authors:** Xinan Yang, Kelly Regan, Yong Huang, Qingbei Zhang, Jianrong Li, Tanguy Y. Seiwert, Ezra E. W. Cohen, H. Rosie Xing, Yves A. Lussier

**Affiliations:** 1 Center for Biomedical Informatics, The University of Chicago, Chicago, Illinois, United States of America; 2 Section of Genetic Medicine, The University of Chicago, Chicago, Illinois, United States of America; 3 Section of Hematology/Oncology of the Department of Medicine, The University of Chicago, Chicago, Illinois, United States of America; 4 Comprehensive Cancer Center, The University of Chicago, Chicago, Illinois, United States of America; 5 Departments of Pathology and of Cellular and Radiation Oncology, The University of Chicago, Chicago, Illinois, United States of America; 6 Ludwig Center for Metastasis Research, The University of Chicago, Chicago, Illinois, United States of America; 7 Computation Institute, Institute for Translational Medicine, and Institute for Genomics and Systems Biology, The University of Chicago, Chicago, Illinois, United States of America; Fox Chase Cancer Center, United States of America

## Abstract

Gene expression signatures that are predictive of therapeutic response or prognosis are increasingly useful in clinical care; however, mechanistic (and intuitive) interpretation of expression arrays remains an unmet challenge. Additionally, there is surprisingly little gene overlap among distinct clinically validated expression signatures. These “causality challenges” hinder the adoption of signatures as compared to functionally well-characterized single gene biomarkers. To increase the utility of multi-gene signatures in survival studies, we developed a novel approach to generate “personal mechanism signatures” of molecular pathways and functions from gene expression arrays. FAIME, the Functional Analysis of Individual Microarray Expression, computes mechanism scores using rank-weighted gene expression of an individual sample. By comparing head and neck squamous cell carcinoma (HNSCC) samples with non-tumor control tissues, the precision and recall of deregulated FAIME-derived mechanisms of pathways and molecular functions are comparable to those produced by conventional cohort-wide methods (e.g. GSEA). The overlap of “*Oncogenic FAIME Features of HNSCC*” (statistically significant and differentially regulated FAIME-derived genesets representing GO functions or KEGG pathways derived from HNSCC tissue) among three distinct HNSCC datasets (pathways:46%, *p*<0.001) is more significant than the gene overlap (genes:4%). These *Oncogenic FAIME Features of HNSCC* can accurately discriminate tumors from control tissues in two additional HNSCC datasets (*n* = 35 and 91, F-accuracy = 100% and 97%, empirical *p*<0.001, area under the receiver operating characteristic curves = 99% and 92%), and stratify recurrence-free survival in patients from two independent studies (*p* = 0.0018 and *p* = 0.032, log-rank). Previous approaches depending on group assignment of individual samples before selecting features or learning a classifier are limited by design to discrete-class prediction. In contrast, FAIME calculates mechanism profiles for individual patients without requiring group assignment in validation sets. FAIME is more amenable for clinical deployment since it translates the gene-level measurements of each given sample into pathways and molecular function profiles that can be applied to analyze continuous phenotypes in clinical outcome studies (e.g. survival time, tumor volume).

## Author Summary

Clinical utilization of multi-gene expression signatures that are predictive of therapeutic response has been steadily increasing, however, interpretation of such results remains challenging because multi-gene signatures, generated from analyzing different patient cohorts, tend to be equally predictive but contain minimal overlap. Whereas pathway-level analyses of expression arrays show promise for generating clinically meaningful mechanistic signatures, current approaches do not permit single-patient based analyses that are independent of cross-group calculations. To bridge the gap between *deterministic biological mechanisms* of single-gene biomarkers and the *statistical predictive power* of multi-gene signatures that are disconnected from mechanisms, we developed FAIME, a novel method that transforms microarray gene expression data into individualized patient profiles of molecular mechanisms. We have validated its capability for predicting clinical outcomes, including cancer patient samples derived from six different clinical trial cohorts of head and neck cancers. This method provides opportunities to harness an untapped resource for personal genomics: clinical evaluation and testing of individually interpretable mechanistic profiles derived from gene expression arrays.

## Introduction

The application of gene signatures to clinical outcome prediction has become an area of intensive research. In cancer, expression signatures of poor prognosis [1], recurrence [2], invasiveness [3], metastasis [4], and therapeutic response [5], [6] have been developed using either data-driven approaches in clinical trials, or via biologically validated mechanisms found prior to the clinical trials. However, gene lists of distinct signatures do not significantly overlap [7], [8], even though they paradoxically occupy a common prognostic space and are similarly efficient in predicting poor clinical outcomes in new cohorts. These observations have *raised questions about their biologic relevance, significance and clinical implication* [7], [8]. New types of mechanism-anchored gene expression signatures are highly desirable for personal genomics but are currently unavailable for single sample prognosis of continuous quantitative phenotypes (e.g. survival time). Since commercial microarrays are now a mature commercial technology and could become a reliable data source amenable to clinical practice, we were motivated to investigate the remaining barriers to their applications in personal genomics.

Aside from differences in computational methods used for deriving gene expression signatures, several hypotheses have been postulated to explain the lack of gene overlap and low reproducibility of the genetic makeup among existing expression signatures. One explanation is that different genes are merely separate aspects of the same groups of molecular pathways or mechanisms [8], [9]. This hypothesis has been examined using the Kyoto Encyclopedia of Genes and Genomes (KEGG) [10] or Gene Ontology (GO) [11] to derive functionally related gene-sets as mechanism-anchored signatures from microarray profiling [12], or from *a priori* knowledge and experimental genome-wide expression data [13]. For conducting such analyses, various analytical and statistical methods have been developed such as DAVID [14], GOstat [15], FunCluster [16], FunNet [17], GSEA [18], MGSA [19], principal component analysis [20], FatiScan [21] and globaltest [22]. These conventional methods of functional gene-set analysis (reviewed in [23], [24]) have improved our overall ability for identifying dysregulated mechanisms from gene expression of a cohort of patients [20], [25]–[29], however they cannot, by design, provide pathway scores at the single sample level. Thus, their potential for clinical usage is limited. Developing the capacity to provide an individualized mechanistic interpretation of analysis results as they relate to clinical outcomes or treatment strategies, will greatly enhance the clinical deployment of signatures.

The state-of-art data-driven but rate limiting methods for generating pathway signatures focus on the coordinated changes in expression of multiple genes in a pathway experimentally detected in animal models [30] or on the knock-in or -down of a key pathway gene in human cells [31], [32]. Recently, two types of knowledge-driven approaches have also been proposed for generating pathway signatures directly from human tumor specimens [33] (a) those using the straightforward unsupervised pathway measures (e.g., mean, median expression of all pathway gene members) within each sample [28], [34], and (b) those generating pathway scores after performing supervised statistics requiring sample class assignment (e.g. principal component analysis, PCA [35]–[37], CORG “condition-responsive genes” [27], LLR [38]). While the latter set of methods is more accurate [27], the *dependencies between samples* preclude their utility for single-sample prognostication. Furthermore, pathway signatures derived from these state-of-the-art methods have been validated in predicting qualitative clinical outcomes, such as *complete remission* vs. *disease progression*. These methods, however, are not designed for making prediction using continuous clinical measures, such as *recurrence-free survival time* [27], [38]. Therefore, novel bioinformatics approaches are required for single-sample assignment of biological features from gene expression analyses so that the wealth of seemingly uninterpretable molecular data can be translated into mechanistic interpretations, which can in turn be utilized for making therapeutic choices and forecasting clinical outcomes.

We hypothesized that molecular mechanisms delineated from gene expression deregulation profiles are accessible as genome-wide measurements of pathways at a single-sample level. Here, we present a novel methodology, the Functional Analysis of Individual Microarray Expression (**FAIME**) that can translate patient microarray data into pathway and molecular functional profiles on a single-sample level and can be applied to quantitative phenotypes of outcome prediction (e.g. survival time, tumor volume response to therapy). FAIME, by computing statistical scores on individual patients, retains sample independence within a cohort and enables subsequent mechanism-level clustering or signature validation. We demonstrate the potential of FAIME in personalized genomics using relatively small-size cohorts of Head and Neck Squamous Cell Cancer (**HNSCC**) in which FAIME produces single-patient survival prediction.

Ninety percent of patients with HNSCC will present with disease that is locoregionally confined and will be considered for curative intent therapy [39]. However, individual outcome prognostication is poor because it is based almost entirely on tumor anatomic location and size [40]. Presently, all patients who are candidates for curative intent treatment are offered a multimodality approach that is associated with serious acute toxicity and long-term dysfunction [41] since there are no reliable indicators to predict response to therapy. Treatment usually consists of broadly cytotoxic entities (e.g. radiation, chemotherapy), and pathobiology based targeted therapies are few [42]. Not surprisingly, we have shown a strong correlation between response to induction therapy and survival [42]. Nevertheless, there are currently no validated pre-treatment classifiers to discriminate the fraction of patients that will benefit clinically from those who will not. Therefore, accurate mechanistic derived signatures would provide valuable prognostic information, the ability to select patients for appropriately intense treatment, and potentially help identify novel targets that could be integrated into current therapy.

## Results

**Figure 1:**
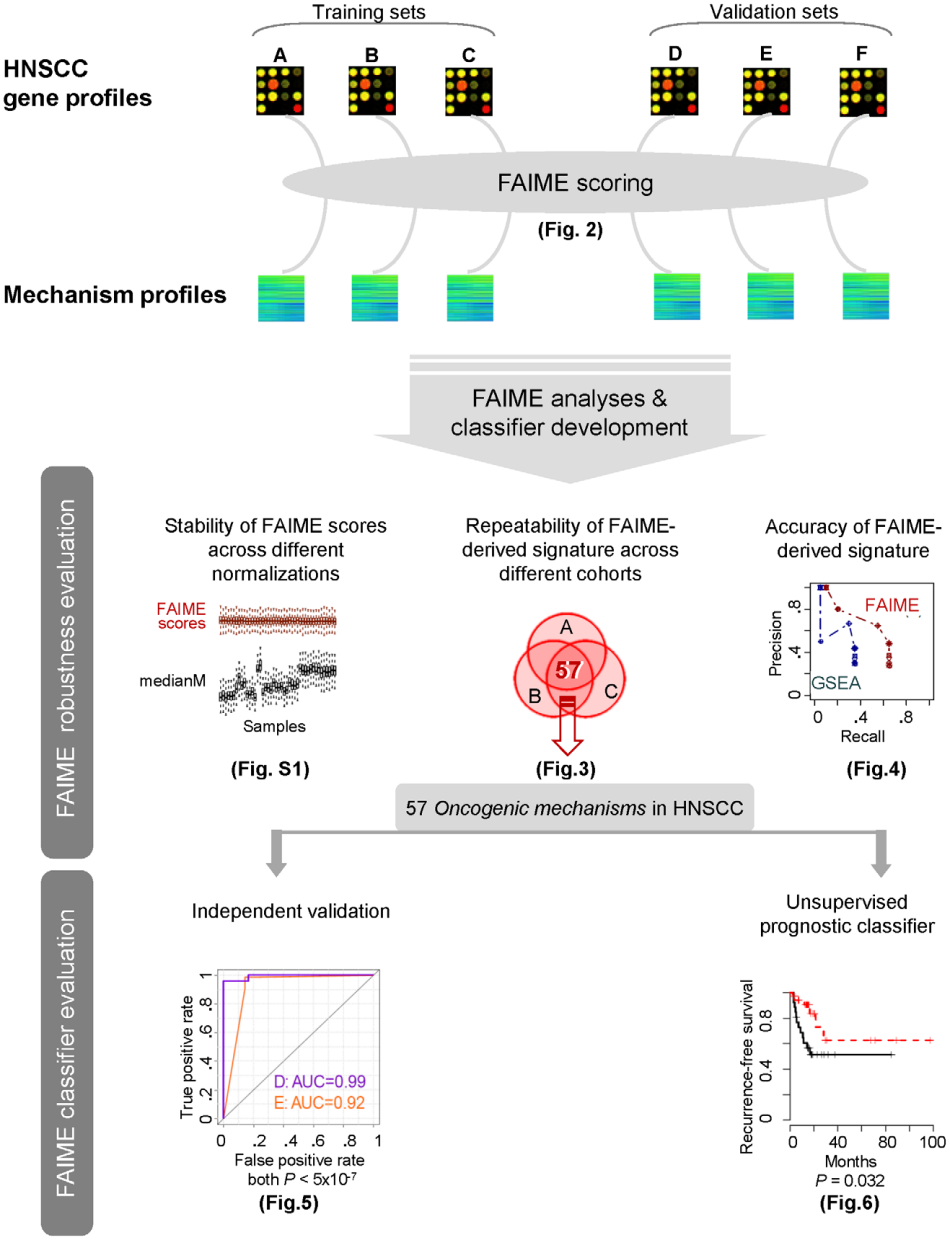
Outline for conducting HNSCC recurrent-free survival prediction using FAIME-derived mechanism profiles of individual samples. FAIME-derived individual mechanism profiles are generated from six independent HNSCC gene expression profiles (Figure 2 in the main manuscript). Then the FAIME Scores are evaluated theoretically for robustness (Figures 3–4, S1) and clinically for new patient diagnosis and survival prediction (Figures 5, S5, 6, S6).

**Figure 2:**
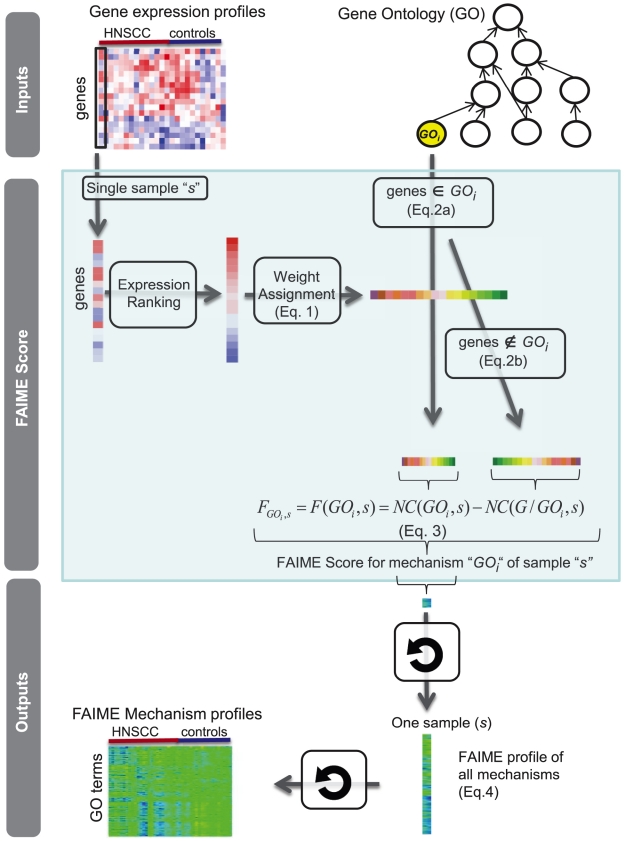
Procedure for determining functional/pathway profiles for each sample using microarray expression (FAIME profiles). The FAIME method is designed to utilize expression arrays of individual samples. Illustrated here with GO terms, this method is also applicable to other functional gene sets such as KEGG pathways. The FAIME profile is sample independent, thus establishing the foundation for truly individualized functional profiles independent of a cohort.

**Figure 1** provides an overview of the experimental design and the main findings of the study. We first evaluate the robustness of the within sample FAIME biological mechanism scores (**Figure 2**) against those of arithmetic mean of all the gene expression values in each pathway (Mean-G) and their median (Median-G). Thereafter, by assigning the clinical group labels to samples, we compare the deregulated biological mechanisms of FAIME Scores (Functional FAIME Score of each gene-set for each sample) across non-tumor control tissue vs. HNSCC tumor samples to those of Mean-G and Median-G as well as those obtained by CORG, a method requiring supervised cross-group calculations for scoring its mechanisms. Taken together, these FAIME-anchored analyses identify oncogenic pathways and molecular functions robustly concordant across three head and neck cancer datasets. Subsequently, these FAIME derived HNSCC feature*s* are used as a multi-mechanism outcome predictor in a straightforward unsupervised diagnosis classification task using independent datasets to demonstrate their predictive capabilities at an entry-level task. Next, the *HNSCC FAIME Feature* features are used for unsupervised prognostic classifiers with the continuous “recurrence-free survival time” variable, which require single sample scoring devoid of class-based assignment to retain independency between classified samples. In order to identify single mechanism outcome predictors, Cox regression analyses are conducted on all individual FAIME mechanism scores in two datasets and two significant prognostic mechanisms are identified by meta-analysis. As a validation, FAIME Scores are compared to the 1^st^ component of a Principal Component Analysis for their prognostication.

### FAIME increases the robustness of scored pathway mechanisms (Figures 3, 4, 5, S1, S2)

**Figure 3:**
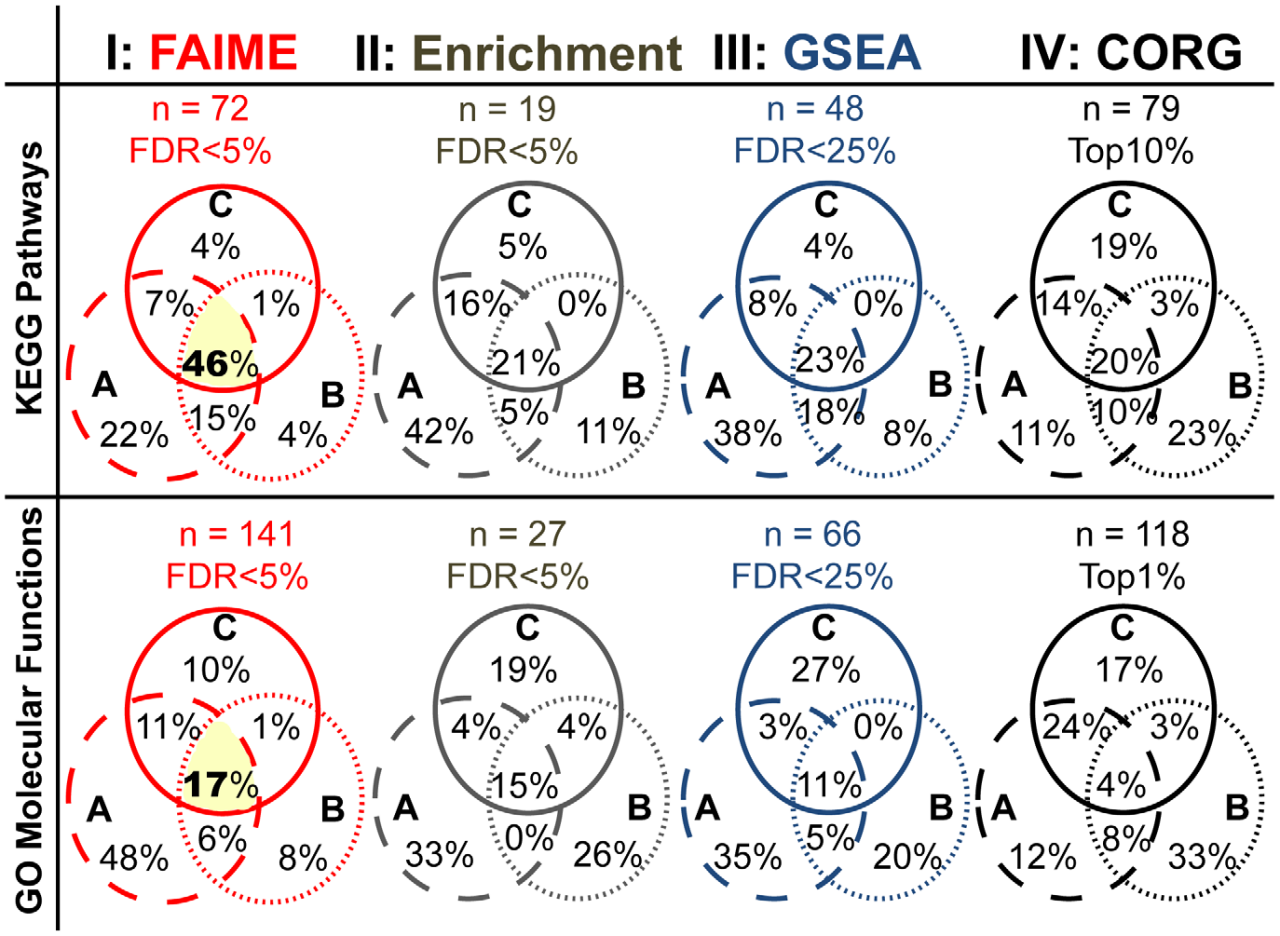
Reproducibility of molecular pathways and functions derived using FAIME as compared to three conventional enrichment methods. To establish that FAIME profiles reliably yield functionally overlapping mechanisms across independent datasets for the same phenotype, we compared the deregulated GO-MF and KEGG FAIME Scores to conventional enrichment studies (hypergeometric enrichment test, GSEA and CORG). The resultant predictions for each method are plotted in their respective columns for both KEGG (top plots) and GO Molecular Function (bottom plots) terms. The letters A, B, and C represent the three analyzed HNSCC microarray datasets (Table 1). Each functional analysis method is independently applied to each dataset and their predicted results are compared in Venn diagrams. FAIME derived features are the most consistent with 47% to 61% KEGG overlap and 18% to 28% GO-MF overlap between any two datasets. In comparison, the next best method in each case provided 23% to 41% KEGG overlap (GSEA) and 15% to 19% (Enrichment). These functional overlapping ranges are well above those generally reported across distinct, yet related, datasets at the gene level [7], [9], [43]. An FDR of 5% is used for FAIME and enrichment results. GSEA is originally reported with a FDR<25% [18] and thus we present its cutoff at a FDR = 25% where overlap is better than for lower FDRs (we observed fewer overlapping GSEA results among the three studies at a 5% FDR). CORG does not report data with p-values or FDRs [27] and the absolute cutoffs presenting the number (n) of resultant KEGG pathways and GO-MF are in a range comparable to that of other methods (Top 10% of KEGG, Top 1% of GO-MF). The 57 Oncogenic FAIME Features of HNSCC (highlighted in yellow, see Methods) that are common to all three HNSCC datasets shown above, 33 KEGG terms (46%) and 24 GO terms (17%) obtained by FAIME (Table S1), are used as seeds for validation studies in three other independent datasets for which the results are shown in Figures 5–6.

**Figure 4:**
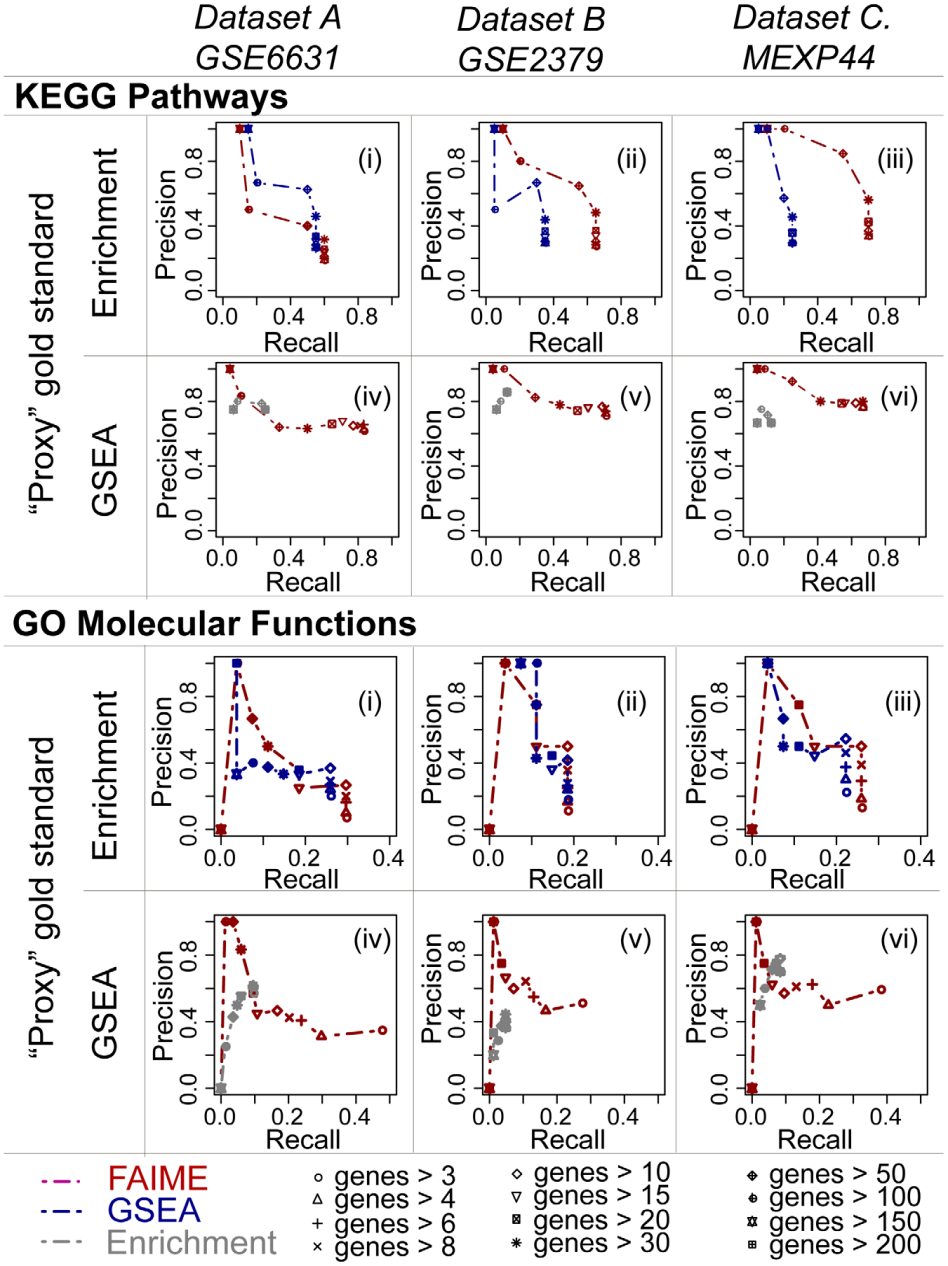
Molecular pathway and function concordance between conventional enrichment methods and differential expression of FAIME profiles. To establish that FAIME profiles yield relevant GO terms and KEGG pathways, we compared the differential expression of these profiles to conventional enrichment studies in three independent HNSCC studies (Figure 3, Table 1, Methods). Enrichment and GSEA studies have been extensively relied upon in biological and clinical studies and are thus alternatively used here as proxy gold standards and positive controls. FAIME-derived significantly altered KEGG and GO-MF (red) of each dataset are compared to those of GSEA (blue) using Enrichment results as a “proxy gold standard” (i,ii,iii in both the KEGG pathway and GO-MF panels). Similarly, these FAIME-derived significantly altered KEGG and GO-MF (red) are also compared to those of Enrichment (grey) using GSEA as the proxy gold standard (iv,v,vi in both panels). The results are reported according to the number of genes annotated in each KEGG pathway and GO term that are detectable on each respective type of array (Table 1). As shown above, FAIME's accuracy is comparable or better than those of the contrasted methods in 10 of the 12 precision-recall curves. Additionally, the high precision of deregulated FAIME profiles between HNSCC tumors and non-tumor control tissues is illustrated against that of a conservative empirical control distribution using pooled GSEA results as a “proxy gold standard” in Figure S3.

**Figure 5:**
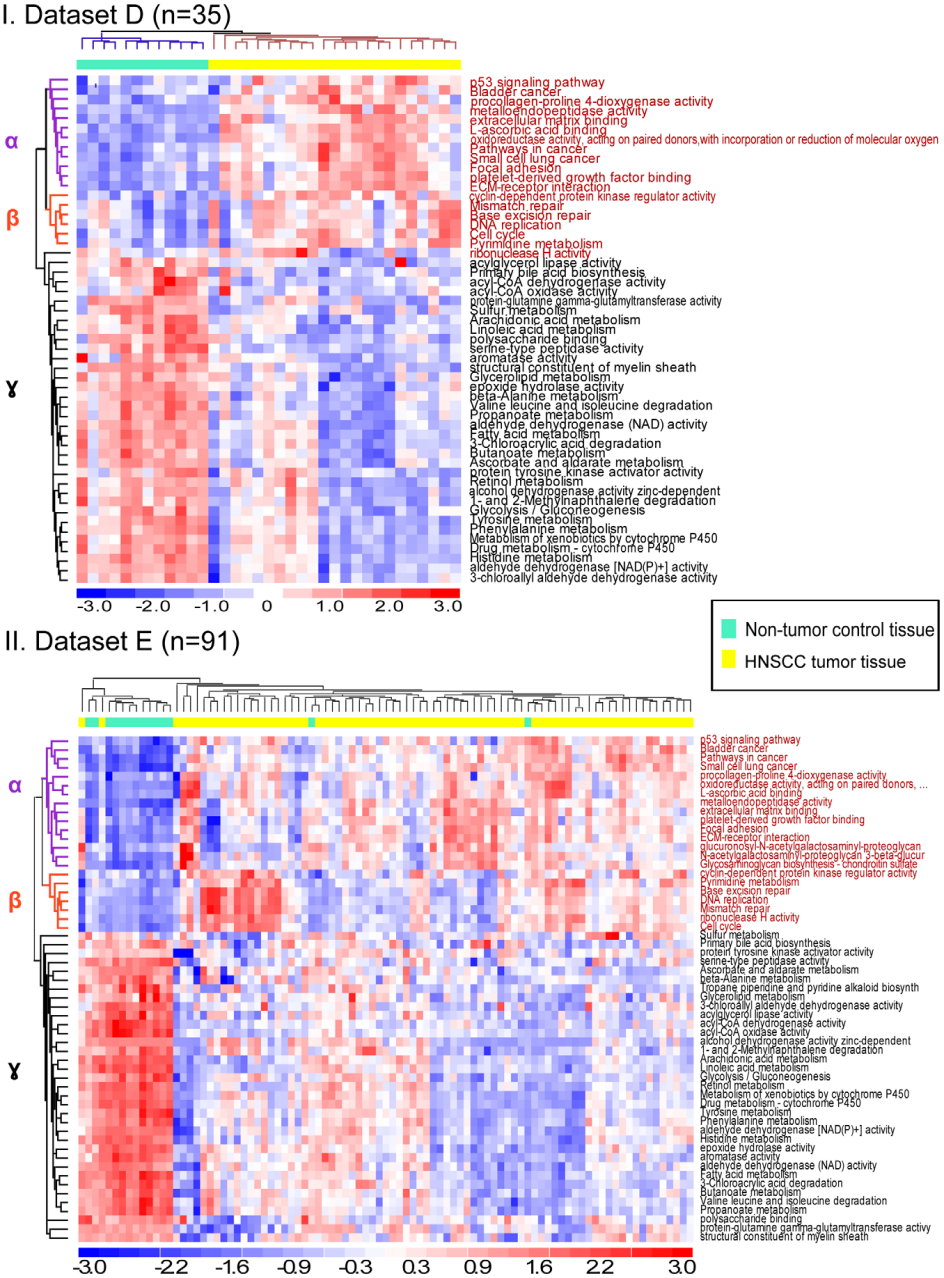
Significantly altered FAIME-derived *Oncogenic FAIME Features of HNSCC* accurately classify samples of two independent validation datasets. In Figure 3, we reported 57 FAIME-derived mechanisms, collectively referred to as the Oncogenic FAIME Features of HNSCC, that were reproduced in each of the independent HNSCC datasets. These consist of 33 KEGG pathways and 24 GO molecular function terms that were significantly deregulated between HNSCC tumor samples and non-tumor control tissue in all three HNSCC datasets at an FDR<5% (A, B and C, Table 1). Here, we demonstrate that the FAIME Scores calculated at the individual sample level in each of the independent validation datasets for these mechanisms can serve as a “functional” profile rather than a gene profile in sample clustering, a distinctive novel property of FAIME. Panels I and II respectively show unsupervised average-linkage hierarchical clustering analysis of the Euclidian distances of FAIME Scores for each sample from the two independent validation datasets, D and E, described in Table 1. As hypothesized, cancer tissue samples are significantly discriminated from non-tumor control tissue ones in both datasets (Panel I: FAIME-Score = 100%, no misclassification, 100 runs of 5-fold cross-validation: AUC = 0.99, p = 4.3×10^−7^; Panel II: FAIME-Score = 97%, 4 misclassifications among 91 samples, 100 runs of 5-fold cross-validation: AUC = 0.92, p = 1.8×10^−12^). Additional conservative empirical p<0.001 are obtained in both panels by permutation resampling of features in AUC calculation. Pathways and molecular functions in sub-clusters α and β (seen on the left of Panels I, II) are also found similarly clustered in both independent evaluations.

The FAIME method provides a translation of each sample's gene expression to molecular mechanisms (FAIME-score). We conduct three analyses to compare the robustness of FAIME to that of previous approaches. We first examine the stability of FAIME Scores within samples, followed by demonstration of the reproducibility of deregulated FAIME-derived mechanisms across three independent HNSCC datasets, and the determination of the precision and recall of FAIME mechanisms using a “proxy gold standard” (**Methods**) as a measure of concordance between FAIME predictions and other methods.

#### Stability of FAIME Scores

In **Figure S1**, FAIME Scores (**first row, panels a–c**) are compared to the unsupervised pathway scoring methods Mean-G and Median-G (**rows 2–3, panels d–i**). These analyses are conducted in HNSCC datasets A, B, and C (**Table 1**) across three preprocessing conditions of gene expression, each with its corresponding column in **Figure S1**. It is obvious that the interquartile spread of FAIME Scores, calculated within samples, is comparable across samples. Compared with those of conventional pathway scoring, FAIME Scores shows higher stability of their pathway scores for every pre-processing condition with a stable median across samples and a slight reduction in variation due to the filtering of genes with low variance (**Figure S1**). Since FAIME Scores are based on the rank of gene expression in each sample, they remain stable when using different gene normalization techniques, and are even applicable to minimalist log-2 transformed gene expression data devoid of cross-sample normalization (**Figure S1, Panel a, Methods**). In contrast, while MAS5 normalization reduces sample-to-sample variability, the distribution of pathway scores derived from the Mean-G or Median-G methods is highly variable across samples. Additionally, the normality of FAIME Scores within samples, termed FAIME profiles are comparable to those of log-transformed gene expression values (**Figure S2**).

**Table 1:** Descriptive summary of six HNSCC datasets.

Dataset ID in present study	A	B	C	D	E	F
Dataset name [reference]	GSE6631 [61]	GSE2379 [62]	E-MEXP-44 [63]	E-MEXP-44_hu6800 [63]	JCO2010 [47]	GSE686 [48]
**Patients and phenotypes**						
Samples (#)	44	38	33	35	91*	60
Control non-tumor tissue (# patients)	22^P^	10	15^P^	12^P^	14	3
HNSCC tumors (# patients)	22^P^	31	15^P^	12^P^	63	55
Cancer recurrence (# patients)	0	0	0	0	8	5
Cervical lymph node metastases (# patients)	0	0	3^P^	11	0	0
Median follow-up time (months)	NA	NA	NA	NA	35	16
**Use of each dataset in our study**						
Identification of *Oncogenic FAIME Features of HNSCC* of HNSCC	**✓**	**✓**	**✓**			
Independent validation (*Oncogenic FAIME Features of HNSCC*)				**✓**	**✓**	
Identification of Recurrence-Free Survival prognosis mechanisms of HNSCC					**✓**	**✓**
**Genomic description of each datasets**						
Expression array platform	Affymetrix HGU95av2	Affymetrix HGU95a/HGU95av2	Affymetrix HGU95a	Affymetrix HuGeneFL	Affymetrix HG-U133 plus2	Agilent Human 1 cDNA microarrays
Genes in the platform	8,799	8,798	8,799	5,408	19,621	7,329
Genes past filtering (**Methods**)	7,764	8,783	8,789	5,349	11,781	4,123
GO molecular functions associated with filtered gene	2,257	2,407	2,408	2,088	2,402	1,629
KEGG pathways associated with filtered gene	216	217	217	214	219	209

**Figure 6:**
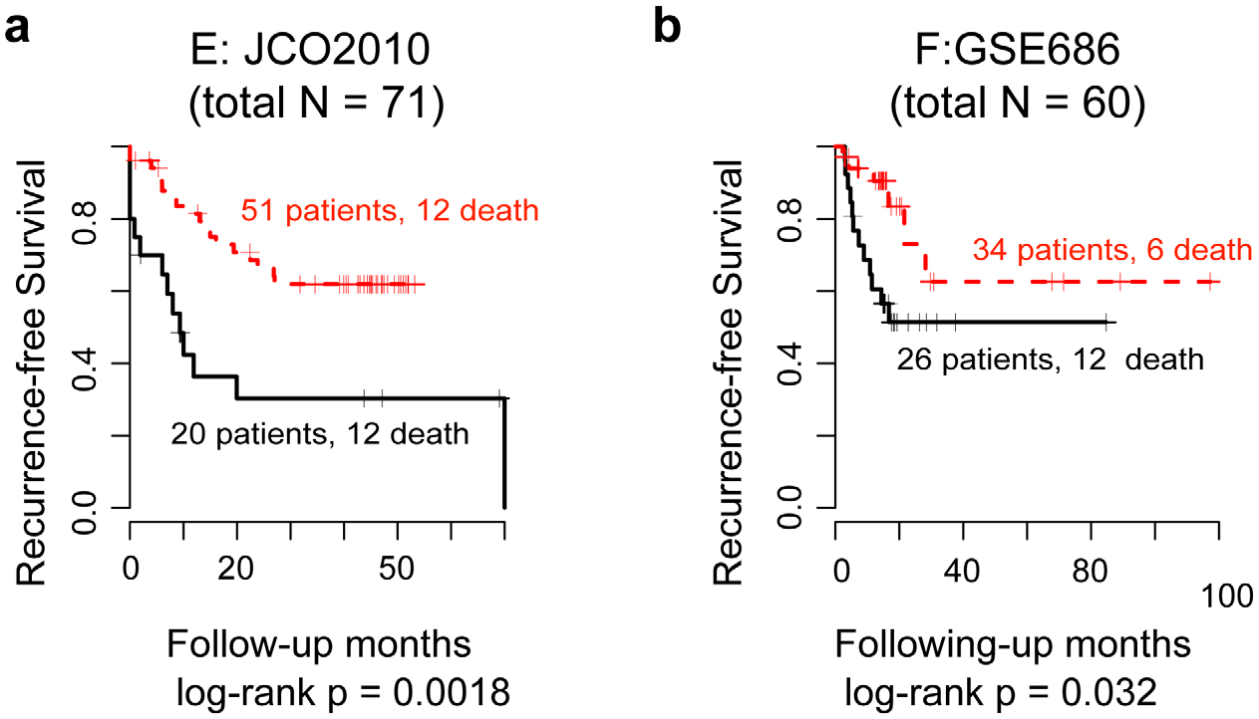
*Oncogenic FAIME Features of HNSCC* stratify recurrence-free survival (Kaplan-Meier) of tumor samples in two independent datasets. The 57 Oncogenic FAIME Features of HNSCC discriminate between HNSCC and non-tumor control tissue at 5% FDR, in each dataset A, B and C, which are independently validated in both datasets D and E (Figure 5). Here, the Oncogenic FAIME Features of HNSCC are analyzed in tumor samples and shown to be predictive of recurrence-free survival as shown in independent datasets E and F (Panels a–b). Oncogenic FAIME Features of HNSCC of each patient tumor sample in datasets E and F are used as a whole with an unsupervised partitioning method to split patients into two groups for which the Cox proportional hazard and Kaplan-Meier curves are subsequently calculated to evaluate the prediction of survival (Methods).

*71 of these HNSCC samples contained survival information and were included for prognosis validation (**Fig. 6**) and all samples were included for diagnosis validation (Fig. 5).

A description of the TNM stage, P53 status, HPV status, smoking status and alcohol intake status is reported in **Table S8A–8C**.

P: paired samples: each patient contributed two samples (one HNSCC tumor sample and one control non tumor oral tissue).

#### FAIME-derived biological mechanisms are reproducible across datasets

In **Figure 3**, the reproducibility of FAIME deregulated mechanisms in HNSCC datasets A, B and C (**Figure 3**, intersection of circles) is compared to that of three other methods. In the analyses of KEGG pathways [10] and of Gene Ontology (GO) molecular functions [11], each sample is assigned to a tumor group or a non-tumor control tissue group. The four methods are thus compared in the two types of canonical mechanisms yielding eight groups of Venn Diagrams, each comprised of three circles, one for each dataset. The intersection of the circles represents overlapping KEGG pathways (top row) and GO Molecular functions (Bottom row), where a high percentage of overlap corresponds to better reproducibility. Conventional methods for determining deregulated mechanisms are (i) hypergeometric gene-set enrichment of reported differentially expressed genes (Enrichment, **Text S1** [14], [15]; **Table 1**) and (ii) Gene Set Enrichment Analysis (GSEA, **Text S1**) [18]. The Condition-Responsive Genes (CORG, **Text S1**) method, which requires supervised group assignment, has been shown more accurate than Mean-G and Median-G in determining deregulated pathways [27] and is thus the preferred choice for evaluation. FAIME obtains the highest reproducibility, yielding 72 predicted GO terms at the union of the three datasets (46% overlap, empirical *p*<0.001; **Methods**). Among these 72 overlapping GO terms, we define 57 ***Oncogenic FAIME Features of HNSCC*** comprising 24 molecular functions and 33 pathways, which are later utilized for FAIME evaluations in classification tasks. In comparison, all three other methods exhibit lower reproducibility of pathway predictions across three data sets (**Figure 3**). Nevertheless, these methods demonstrate higher reproducibility at the molecular mechanism space (15–23% overlap) when compared with 4% direct gene overlap among the same datasets [7], [9], [43]. In addition, as shown in **Table S1**, a significant portion of the 57 *Oncogenic FAIME Features of HNSCC* are also identified by either enrichment, GSEA, or CORG (93%) or by two out of the three methods (65%). A more thorough evaluation of FAIME's concordance with alternative state of the art methods is also conducted and discussed as below (**Figure 4**).

#### FAIME-derived biological mechanisms are concordant with those obtained by conventional enrichment methods

**Figure 4** illustrates that the biological mechanisms predicted by deregulated FAIME Scores are reliably concordant with those of our “proxy” gold standards (conventional Enrichment and GSEA methods) in each dataset A, B and C. These KEGG pathways and GO molecular functions previously derived by each of these methods (**Figure 3**) are compared across methods in each dataset individually, rather than across datasets, to yield accuracy scores (**Figure 4**). FAIME predicted 60, 46 and 41 KEGG Pathways and 116, 45, and 55 GO Molecular Functions in datasets A, B, and C respectively totaling 72 and 141 distinct pathways and molecular function predictions (see high reproducibility of FAIME in **Figure 3**). The “proxy” gold standard for each of the precision-recall curves corresponds to one of the two conventional enrichment methods, alternated accordingly, allowing us to compare the accuracy of FAIME predictions to those of one of these methods (left column of **Figure 4**). For example, the topmost right area of **Figure 4** **-Panel (iii)** uses the KEGG pathway mechanisms predicted by enrichment in dataset C as a proxy gold standard to evaluate the precision and recall of GSEA predictions (blue line) and those of FAIME (red line). Subsequently GSEA serves as a proxy gold standard in **Figure 4** **-Panel (vi)** to evaluate the predictions from enrichment (grey) and those of FAIME (red). Overall, the accuracies of the FAIME derived mechanisms perform at comparable or better levels than those produced by conventional methods in 10 out of the 12 precision-recall curves, confirming its higher reproducibility in comparison to the well validated conventional methods. In **Figure S3**, we also construct an extensive empirical distribution of KEGG pathways and GO Molecular Functions predicted by FAIME as a control to conservatively confirm the significance of the observed overlap reported in **Figure 4**.

#### *Oncogenic FAIME Features of HNSCC* accurately classify samples from two independent datasets and provide biological insights into head and neck cancer (Figure 5, Table 2)

**Table 2:** Robustness of the accuracy of FAIME-derived *Oncogenic FAIME Features of HNSCC* in separating tumor from control tissue samples.

Measure of Accuracy →		FAIME-Score			
*Oncogenic FAIME Features of HNSCC* →		24 Molecular Functions (GO-MF)§		33 Pathways (KEGG)§	
Clinical Datasets (see Table 1: D, E) →		D	E	D	E
Unsupervised Clustering method*	Software				
Ward linkage of Euclidean distances	Bioconductor package (*amap*)	100%	98%	96%	97%
Average linkage of Euclidean distances	dChip	100%	98%	98%	97%
Centroid linkage of Correlation distances, with column standardization for sample clustering	dChip	100%	98%	98%	96%
Centroid linkage of Correlation distances	dChip	100%	98%	98%	96%
Partitioning Around Medoids (PAM, k = 2)	Bioconductor package (*cluster*)	100%	96%	96%	96%

57 deregulated Oncogenic FAIME Features of HNSCC were reproducibly derived by FAIME in the three independent datasets (A, B, and C; **Figure 3**, **Table 1**) and shown that they can jointly classify non-tumor control tissue from HNSCC tumors in two validation datasets (D and E; **Figure 5**, **Table 1**). This table also illustrates that the constituents of the HNSCC Oncogenic FAIME Features of HNSCC (columns), the 24 GO Molecular Functions and the 33 KEGG pathways, were also independently accurate in separating tumor from control tissue samples in the validation datasets. Furthermore, we report that this predictive accuracy is robustly reproduced using five different unsupervised clustering algorithms (rows below). In the remainder of the manuscript, these Oncogenic FAIME Features of HNSCC are pooled together for prognosis predictions in **Figures 5** **–** **6**.

*Default parameters of the clustering algorithms were used unless otherwise specified. After applying each clustering method to the *FP_HNSCC,s_*, the first two-way partition of samples was used to determine two classes compared with the tumor sample group and the control group of two independent datasets D and E (**Table 1**). False positive results were control samples misclassified within the tumor sample cluster and false negative results were tumor samples classified as control tissue. The FAIME-Score accuracy for separating 23 HNSCC tumors from 13 control samples of the E-MEXP44_hu6800 array was calculated as 
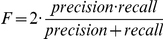
.

**§:** : GO-MF terms and KEGG pathways were not included if there were no mapped genes that passed the IQR filter in this validation dataset.

**Legend**: *FP* ***_HNSCC,s_*** is defined as the subset of the Functional FAIME Profile consisting of the 57 *Oncogenic FAIME Features of HNSCC* deregulated in the initial datasets A, B and C (**Equation 4**, Methods, **Figure 3**); GO: Gene ontology, MF: molecular function.

In order to confirm the capability of the *Oncogenic FAIME Features of HNSCC* to discriminate HNSCC phenotypes from non-HNSCC, and to assess the clinical relevance of FAIME Scores, we conduct a retrospective analysis of two independent HNSCC datasets. The 57 *Oncogenic FAIME Features of HNSCC* are able to classify clinical samples in independent datasets D (n = 35), E (n = 91), and GSE9844 (n = 38) with zero, four, and three total misclassifications, respectively (**Figure 5** **, Figure S4**). Furthermore, samples from the two validation datasets can be accurately classified by the 24 molecular functions from GO (GO-MF) subset, or by the 33 KEGG pathways subset of the *Oncogenic FAIME Features of HNSCC* as shown in **Table 2**. The classification power of these *Oncogenic FAIME Features of HNSCC* is robust as it is confirmed by four conventional unsupervised classification methods tested (**Table 2**, **Methods**). When used jointly, the FAIME prioritized GO-MF and KEGG pathways are equally successful in separating HNSCC tumor samples from non-tumor controls in datasets D and E (Dataset D: n = 35, area under the curve (AUC): AUC = 0.99, *p* = 4.3×10^−7^; Dataset E: n = 91, AUC = 0.92, *p* = 1.8×10^−12^, **Figure 5**). The detailed accuracy metrics show that the FAIME Scores are comparable for classifying tumor vs. non-tumor control to those of gene-based classifiers used in head and neck cancer (**Table S2**).

As shown in both panels I and II of **Figure 5**, genes in sub-clusters α and β of up-regulated KEGG pathways and GO molecular functions are consistently classified together in both validation datasets D and E by unsupervised methods. The reproducibility of these sub-clusters suggests a biological modularity of their underpinning mechanisms (full list detailed in **Table S1**). Cluster α consists of a set of intra- and extra-cellular events such as cancer pathways, p53 signaling, ECM-receptor interaction and focal adhesion. Cluster β consists of nuclear events that control DNA replication, DNA damage repair, and cell cycle progression. Although each individual mechanism of Clusters α and β is known for their involvement in the development of human cancer, the observed coordinated activation of all components within each cluster is novel. It suggests that oncogenesis requires not only one pathway activation or molecular function enhancement, but also the cooperation among a majority or all of the mechanisms we have prioritized. Additionally, we have also identified a third cluster – (Cluster ϒ, **Figure 5**) which consists of 35 KEGG pathways or GO molecular functions that exhibits coordinated down regulation in tumor samples of both validation datasets D and E. They collectively indicate a reduction in a broad spectrum of biosynthesis events and metabolic activities (**Table S1**). There is an increasing appreciation and understanding of the involvement of metabolism in cancer. Our observations of the extensive and coordinated reduction in cellular biosynthesis and metabolism support the notion that cancer is a “metabolic disease” [44], [45].

In summary, the application of the FAIME algorithm to multiple scales of biological functions (pathways, molecular functions) demonstrates a novel quantitative approach that is capable of identifying distinct molecular mechanisms associated with the onset of cancer, and could thus facilitate the elucidation of complex biology.

### *Oncogenic FAIME Features of HNSCC* predict recurrence-free survival (RFS) of new patients (Figures 6, S6)

In addition to its ability to discriminate between the non-tumor control and HNSCC tumor tissues, the 57 *Oncogenic FAIME Features of HNSCC*, as a whole, have significantly higher prognostic power. They stratify HNSCC samples in two distinct HNSCC datasets (E and F) into two recurrence-free survival subgroups (log-rank *p* = 0.0018 and 0.032, dataset E and F, respectively; **Figure 6**). This survival analysis exemplifies a distinctive task that FAIME was designed to accomplish: sample's mechanisms scores are calculated without group assignment and thus circumvent the risk of overtraining. We further validate the prognostic power of FAIME to predict survival of patients from an additional HNSCC dataset previously not used in this study, GSE2837 (Log-rank *p*  = 0.049, Kaplan-Meier curve in **Figure S5**). We then compare the utility of FAIME-derived scores with other enrichment methods that also do not require phenotypic group assignment. In contrast, Enrichment analysis, Mean-G and GSEA scores fail to provide stratification of patients by RFS time (*p*>27%, *p*>7%, and *p*>34%; smallest log-rank *p* reported from dataset E or F; Enrichment, Mean-G and GSEA respectively; **Methods**). These results attest to the utility of FAIME for learning predictive mechanism patterns from gene expression in pursuance of quantitative phenotypes such as survival analysis. In order to compare the prognostic power of FAIME to other prognosticators of HNSCC, we note that for dataset E in this study, the authors report a non-statistically significant trend for HPV+ HNC patients to have better survival outcomes when compared to HPV- HNC patients (HNSCCs classified according to Pyeon HPV geneset [46]: *p* = 0.09 and *p* = 0.33 for two independent datasets; HNSCCs classified HPV+/− by p16 IHC: *p* = 0.20 and *p* = 0.52 for two independent datasets). In contrast, we show that when FAIME is run on this dataset, we obtain a significant prognostic indicator for survival outcome for datasets E, F, and GSE2837 (*p* = 0.0018, *p* = 0.032, *p* = 0.049, respectively) in **Figure 6** **and Figure S5**.

We next investigate if these FAIME-derived patterns could recapitulate known biological and pathophysiological knowledge. As shown in **Figure S6**, six of the 57 *Oncogenic FAIME Features of HNSCC* significantly overlap with the KEGG pathways and GO-MF that can be derived by enrichment of the 31 genes (54 probes) associated with disease-specific survival in a study by Thurlow et al. [47]. In the second study by Perou and colleagues, a set of 582 deregulated genes has classified HNSCC into four “intrinsic” groups (I–IV), of which some combinations are associated with poorer recurrence-free survival [48] (dataset F, **Table 1**). Importantly, FAIME can recapitulate at the mechanism level this molecular classification: by producing four groups using an unsupervised method, the 57 *Oncogenic FAIME Features of HNSCC* are enriched in the four original “intrinsic” groups of Perou's molecular classification of HNSCC samples (*p* = 0.0031, **Methods**, Dataset F, Fisher's Exact Test on a 4×4 contingency table).

It has been demonstrated that some multi-gene expression signature classifiers, derived from comparisons made between the cancer and control tissues, can provide both diagnostic stratification of clinical tumor samples as expected, as well as prognostic prediction of clinical outcomes, such as RFS or overall survival across patients within a clinical cohort [47], [48]. However, to our knowledge this is the first mechanism-level predictor, generated from gene expression changes between the cancer and control tissues, that possess both diagnostic and prognostic power at the level of each individual clinical sample without requiring group assignment in validation sets. These observations also indicate that common molecular mechanisms may underlie oncogenesis and disease recurrence. Therefore, therapeutic targeting of such common mechanisms may have potential clinical benefits of effective local control of primary tumors, and at the same time, preventing disease recurrence.

### FAIME-derived single mechanisms also predict recurrence-free survival (RFS)

In order to prioritize other potential RFS mechanisms and to further demonstrate the utility of FAIME Scores for the study of quantitative phenotypes (e.g. RFS), we conduct a Cox regression analysis of recurrence-free survival time for each of the 208 KEGG pathways and 956 GO terms for which a FAIME Score could be computed in datasets E and F with four or more genes per mechanism (**Methods**, **Table 1**). Two significant mechanism genesets can each be considered as a “single mechanism” prognostic predictor of RFS for future clinical validation: (i) [hsa04210], and (ii) *receptor signaling complex scaffold activity* [GO:0030159] (*p* = 0.0026 and 0.0034 respectively; Bonferroni-adjusted meta-analysis across Datasets E and F of the Cox regressions **Table S3**, **Methods**). Since disease recurrence is also a predictor of RFS, FAIME Scores of these pre-treatment samples are also significantly decreased in patients with rapid onset of disease recurrence in both datasets E and F (**Table S3**, Mann-Whitney test).

As a proof-of-concept study we focus on the topmost candidate RFS mechanism, and conduct a Principal Component Analysis (PCA) of datasets E and F for the prioritized KEGG apoptosis geneset (KEGG pathway [hsa04210] containing known genes associated with apoptosis pathways annotated in humans) since PCA can provide an unbiased approach to derive a metric representing the highest biological variation across samples that can be associated with the biological mechanism(s) responsible for this variation. As expected, PCA's first component of the KEGG apoptosis geneset gene expression is also a predictor of deregulated RFS (Cox regression *p* = 0.0027 and 0.044, Datasets E and F). Furthermore, this first component and the FAIME Scores of the KEGG apoptosis geneset are correlated (Spearman *p* = 1.3×10^−8^ and 0.047 in datasets E and F respectively, **Table S3**). The detailed list of genes of the KEGG canonical apoptosis geneset is listed in **Table S4**. Additionally, we show that FAIME Scores of the KEGG apoptosis geneset are significantly increased in the HNSCC tissues of patients with no evidence of disease recurrence as compared to those of patients with recurrence within both datasets E and F (*p* = 0.0015 and *p* = 00051, respectively, **Figure S7**). Consistent with these results, HNSCC patients are treated with radiotherapy and conventional chemotherapy regimens that should subsequently induce apoptosis through DNA damage cell cycle checkpoints. Therapy-resistant tumors are more likely to recur early, which is consistent with the observed reduction in the FAIME apoptosis geneset score in patient samples with disease recurrence reflecting a reduced capability of checkpoints to elicit an apoptotic response to therapy-induced DNA damage. The correlation of reduced level of expression of the KEGG apoptosis geneset reported by the FAIME Score in patients with more rapid onset of disease recurrence may reflect aberrant cell cycle checkpoint and DNA damage repair regulation leading to an enhanced survival of HNSCC cancer cells in recurrent patients. This computational FAIME method identifies deregulated genesets associated to mechanisms, and after validation of the FAIME Scores in a prospective study, we will also pursue to validate the deregulation of mechanisms associated to this genesets, such as the apoptosis geneset. Since a pathway-level classifier can be considered as a significant predictor based on the ensemble effect of its constituent gene in a patient, these same genes need not be consistently deregulated in each patient. In other words, the effect is measured at the mechanism level.

## Discussion

FAIME Scores are designed to identify molecular mechanisms whose constituent genes are predominantly up- or down-deregulated, but not both together. We thus regard the present design as a stepping-stone that improves clinical and biological interpretability by reducing the number of features as compared to gene expression signature classifiers, and by obtaining increased statistical power that allows for the inclusion or refutation of each mechanism at the single patient level. Alternatively, the gene expression changes in both directions could be assessed indiscriminately so that subtle changes in molecular mechanisms due to the opposite effect of inhibitor and activator genes of a given pathway can also be identified. Better still, pathway annotations that indicate gene inhibitors and gene enhancers of signals could be pooled together according to the logical direction of the biological significance in the pathway.

In principle, FAIME Scores are applicable to other scales of individual quantitative genomic data annotated into genesets from a knowledgebase, for example, from single protein activity measurements. For these extensions, alternative decreasing weights and their effects on FAIME Score need to be discussed. In this manuscript, the weighting strategy used by the FAIME Score is a rank based decreasing from the highest expressed genes to non-expressed genes in individual samples. We chose the weights to decay exponentially based on previous modeling of expression data we conducted [49], [50] (**Equation 1**), arguably future studies may explore alternate models, particularly for different types of genomic data that have not been modeled.

Currently, the effort required for interpreting the biological significance of individual genes in expression signature classifiers is challenging. As shown by Bild et al, mechanism-based predictors can bring us one step closer to guiding targeted therapies by using oncogenic pathways derived from cellular experiments [35]. Additional improvements are required to translate FAIME technology for its use in a clinical setting. Specifically, we intend to prospectively validate the capability of *Oncogenic FAIME Features of HNSCC* to predict survival in a cohort of HNSCC patients treated with a) cytotoxic chemotherapy (e.g. induction chemotherapy), b) radiation, and/or c) an EGFR inhibitor. Future studies will test FAIME's ability to distinguish between patient tumor subtypes: 1) HPV positive vs. HPV negative, 2) low risk for failure vs. high risk for failure to therapy, and 3) emerging genetic drivers of HNSCC (ongoing TCGA cancer genome atlas effort in head and neck cancer). Here, we describe the development and evaluation of FAIME, a computational rather than biological approach designed specifically with the intent for enabling clinicians to functionally interpret individual patient samples using quantitative phenotypes. Additional improvements are required to translate this technology into clinical care, such as the ability to directly interpret a single microarray without cross sample normalization. For example, the “Gene Expression Barcode” relies on a reference standard to interpret single array gene expression [51], [52]. Furthermore, we and others have shown analytical approaches that do not require a reference standard nor cross-sample normalization to interpret virus genesets at the single pan-microbial array level [53], [54] whose clinical utility has been documented in some case reports and studies [55], [56]. In principle, these approaches could be used jointly with FAIME to improve the efficiency of single sample analyses. We note, however, that our analysis of differential gene expression and corresponding mechanisms of head and neck cancer may be complicated by the process of “field cancerization,” whereby an area of epithelium is preconditioned by a carcinogenic agent, and carcinoma may subsequently arise from multifocal areas [57]. Thus, future expression analyses should scrutinize obtaining non-tumor tissue controls as to avoid the effect of “field cancerization” by using matched controls. Additionally, testing datasets analyzed in this study obtained gene expression data from Affymetrix and Agilent platforms, confirming that our approach is amenable to different gene expression platforms and future studies may utilize additional tissue arrays.

Indeed, we recognized that different extraction methods of the tissue, including laser microdissection, could contribute to generating different classifiers. Conceivably, classifiers built from non-laser microdissected data may reveal important genetic signatures encompassing tumor interactions with surrounding stromal cells, but more precise methods are needed to isolate these cells. We note that all tumor samples in the three training datasets and testing datasets D, GSE2837, and GSE9844 contain at least 70% tumor cells (**Table S5**). We provide in **Figure S4** showing the validation of the *Oncogenic FAIME Features of HNSCC* in an independent laser microdissected tumor and non-tumor tissue dataset GSE9844 with a positive predictive value = 92% (3 misclassifications) and *p* = 0.04 (rank statistics, 100 feature permutations, hierarchical clustering analysis using R). Furthermore, in our comparison of laser microdissected tumor tissue with non-tumor control tissue (GSE9844), we identified 751 out of 2,699 significantly deregulated FAIME Scores that were calculated using genesets of mechanisms (q-value<0.05, z-test adjusted for multiple comparisons). Of the 57 *Oncogenic FAIME Features of HNSCC* (57 GO and KEGG genesets), 47 were confirmed among these 751 and are deregulated in the same up or down directions originally observed in datasets A, B, and C (*p*<10e-16, enrichment study using Fischer Exact Test, **Table S6**). Furthermore, we found that *Oncogenic FAIME Features of HNSCC* were able to accurately classify laser microdissected tumor tissues from non-tumor tissues (GSE9844, n = 38, 3 misclassifications using conventional unsupervised two-way hierarchical clustering). We have thus demonstrated that utilizing FAIME Scores to classify laser microdissected tumor tissue is not only feasible, but is also concordant with results from non-laser microdissected tissue datasets in this study.

Conventional pathway level classifiers obtained from *in vitro/in vivo* biological experiments are predetermined and rate limiting for discovering multiple oncogenic pathways or mechanisms, as each require their own biased experimentation [31], [32], [35], [58]. In contrast, *in silico* knowledge driven approaches are high throughput and can analyze, in an unbiased manner, a larger number of biological mechanisms. Improved reproducibility is achieved with machine-learned mechanism-based predictors that use group assignment (e.g. CORG [27], LLR [38]), as compared to mechanism predictors derived from the straightforward scoring methods such as Mean-G and Median-G. While “group assignment”-dependent methods are effective in imputing mechanisms for qualitative phenotypes, they are not designed for imputing from a quantitative phenotype. We have designed FAIME to address this challenge. We show that a FAIME-derived predictor composed of *Oncogenic FAIME Features of HNSCC* can withstand regression analyses of continuous survival time data to predict disease free survival. Beyond predictor construction, we have shown that FAIME-derived mechanisms controlled for multiple comparisons can be retained independently as single mechanism classifiers. We also demonstrate that *Oncogenic FAIME Features of HNSCC* can classify reproducibly head and neck cancer survival, and have the potential for new knowledge discovery. Furthermore, this approach can conservatively prioritize three classifiers based on a single mechanism classifier for experimental or clinical follow-up validation. Future studies will address FAIME's applicability to a variety of other cancers and tissue-oriented diseases, including oncogenic FAIME Scores of colon cancer metastases progression and of prostate cancer recurrence that are underway.

Survival in HNSCC is currently assessed with a mix of TNM staging and biomarker status, such as Human Papilloma Virus (HPV). The utility of gene expression signatures in HNSCC lags behind better suited ones developed in other cancers, such as breast cancer, where expression classifiers are commercially available (e.g. MammaPrint® microarray [59]. **Table S2** shows that the accuracy of *Oncogenic FAIME Features of HNSCC* to classify tumor vs. non-tumor tissue is comparable to or outperforms those of gene expression classifier signatures in head and neck cancers from the respective papers. However, gene expression signatures are not utilized in clinical settings for head and neck cancers, in part because of their lack of genetic overlap. Deregulated GO and KEGG FAIME Scores have shown a significantly higher overlap between datasets (**Figure 2**) than deregulated genes between these datasets (Legend of **Figure 2**). This reproducibility of FAIME Scores addresses a crucial problem of gene-level expression signatures illustrated by Dr. Joan Massagué in an editorial of the New England Journal of Medicine [60]. In summary, Massagué points out that gene signatures have surprisingly very little overlap when designed in different cohorts, even though they may be equally predictive of the same clinical outcome in these cohorts. In the future, the survival prediction of FAIME Scores is expected to help identify patients, among those for which traditional clinical indicators are inadequate, at high risk of treatment failure that would benefit from more intensive therapy.

## Methods

### Data preparation and databases

Eight **gene expression microarray datasets** pertaining to HNSCC are used: three for learning expression patterns and for demonstrating concordances of FAIME (**Table 1**: datasets A–C [61]–[63]), while five other datasets are used for validation (**Table 1**: datasets D–F [63], [47], [48], **Figure S4** laser-microdissected dataset GSE9844 [64] and **Figure S5** dataset GSE2837 [65]). The samples of the validation datasets do not overlap with the learning datasets. We define **non-tumor control samples** as (i) samples from an independent, non-smoker individual with no history of HNSCC, (ii) paired samples from a distant uninvolved site in patient with HNC (>3 cm or contralateral), and (iii) paired samples from the margin of a tumor. For each of these three types of control samples, **Table S7** provides the percentage of samples per anatomical location. Several of the non-tumor tissue control samples are enriched specifically for epithelial cells (datasets B, E, F, GSE2837, GSE9844), the cell type of origin for head and neck cancer cells, while other non-tumor controls were extracted from mucosae tissues (datasets A, C and D). Additionally, a description of the TNM stage, P53 status, HPV status, smoking status and alcohol intake status is reported in **Table S8A–8C**.

#### Pre-processing of gene-expression profiles

The gene expression CEL files of the GEO and ArrayExpress datasets A–D of **Table 1** are normalized by the MAS5 method using the Bioconductor package *affy*. The normalized gene expression profiles of the larger datasets E and F (**Table 1**) are directly downloaded from GEO and all measurements are log2-transformed. **Probe-set filtering**. Since probe-sets with little variability across samples provide no discriminatory power [66], the probe-set of a gene with the largest inter-quartile range (IQR) of gene expression is retained for this gene in the study after removing genes for which the average log2 expression across samples is negative (**Table 1**). As such, filtering procedure focuses on the most informative genes and can control for multiple probe-sets per gene bias.

**Microarray platform annotation** was downloaded from the GEO website (http://www.ncbi.nlm.nih.gov/geo/) for the GSE686 dataset using an Agilent platform. For the other datasets using Affymetrix platforms, the annotations are derived from the Bioconductor package *hgu95av2.db_2.3.5*, *hu6800.db_2.3.5* and *org.Hs.ef.db_2.3.6*.

**Gene Ontology (GO) annotations** for human genes were downloaded from NCBI (ftp://ftp.ncbi.nlm.nih.gov/gene/DATA/gene2go.gz) on December 11, 2009. The GO Molecular Functions (GO-MFs) with more than 3 annotated genes are studied.

**KEGG pathway annotations** are imbedded in Bioconductor database *KEGG.db* version 2.3.5. This map is based on data provided by: KEGG GENOME (ftp://ftp.genome.jp/pub/kegg/genomes) with a date stamp of September 16, 2009. The KEGG pathways with more than 3 annotated genes are studied.

### FAIME profile (Figure 2)

#### Computes the normalized centroid of each mechanism-anchored gene-set based on the rank-weighted gene expression of a sample

The FAIME profile is designed to utilize expression arrays of individual samples for establishing the basis of generating individualized functional profiles (**Figure 2**) that can be directly used to identify gene-sets that separate patients into clinical groups or other clinically useful continuous variables such as survival time or tumor volume. The following section describes the steps used to calculate the “functional” profile of FAIME (FAIME profile). For brevity, we illustrate mechanism-based FAIME Profiles in the following equations using GO terms, while KEGG-annotated FAIME profiles are calculated similarly.

To quantitatively assign a mechanism's “expression deregulation” via its gene members, whose expression is measured in a microarray, all expressed genes (set ***G***) in each sample are sorted in a descending order according to their expression levels, and then an exponential decreasing weight (*w*) is assigned to the ordered genes (**Equation 1**). The resultant weighted expression values are used to prioritize relatively highly expressed genes as in the first step of Bioconductor package *OrderedList* [49], [67]. Specifically, let ***r_g,s_*** be the expression rank for each gene ***g*** ∈ ***G*** in a sample ***s***, let |***G*** **|** be the total number of distinct genes in the array and the weight assigned to each gene per sample (***w_g,s_***) is calculated as follows:
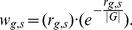
(1)


A Normalized Centroid (***NC***) is defined as the uni-dimensional average of the weighted expression values of a gene-set. Specifically, the sum of the weighted expression of gene element in a gene-set is normalized according to its cardinality. For every GO term, there is a gene-set ***GO_i_*** in which genes satisfy *g* ∈ *GO_i_* and a complement gene-set (G/GO***_i_***) comprised of all available genes in the array that are not annotated to this GO term. Thus we calculate the normalized centroid of each gene-set *GO_i_* in each sample ***s*** and that of its complement gene-set as follow:
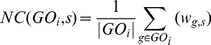
(2a)

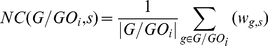
(2b)

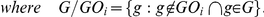



Furthermore, the Functional FAIME Score (***F in equations***) of each gene-set of a GO term is calculated in every sample as the difference between the normalized centroid of its gene-set and that of its complement gene-set (**Equation 3**). We define functional scores as functional biological mechanisms of the gene-set associated with a GO term in a given example.
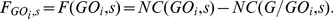
(3)


**Equation 4** calculates for a sample *s*, the **FAIME Profile** “***FP*** *_s_*” defined as the set of all of FAIME FAIME-Scores of sample *s*, 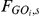, assigned to every GO term.
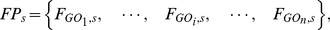
(4)where ***n*** is the total number of GO terms.

In this way, patient-specific FAIME profiles of KEGG and GO are generated for each sample (**Figure 2**). Each sample has a continuous effective value for each category term which is the group difference between the genes annotated by the KEGG or GO terms and their individual complementary set of genes [50].

#### FAIME Profiles of HNSCC microarray data

For each microarray dataset (**Table 1**), gene expression is transformed into FAIME profiles of KEGG pathways and GO molecular functions for each individual sample in all datasets (A–F).

FAIME scoring and profiling are implemented using open source R software (**Text S2**).

### FAIME score stability, robustness and reproducibility evaluations

#### Stability and robustness of FAIME scores with and without cross-sample normalization and gene filtering (Tables 1–2, Figure S1)

To evaluate the stability of FAIME-derived pathway scores (**Eq.3**) regardless of the gene expression preprocessing methods, a FAIME Score distribution for each sample is illustrated using boxplots (**Figure S1**). These boxplots present the median, 25% to 75% interval of distribution (box) and the 1.5 inter-quartile ranges (dashed vertical whiskers) calculated using R package *graphics*. Specifically, the FAIME-derived pathway scores are applied to (i) minimalist within-sample log_2_ transformation, (ii) within- and across-sample normalization using MAS5 method [68], and (iii) MAS5 normalization with gene filtering (see **Data preparation and databases**). Using the “*affy*” package [68] of the Bioconductor software [69], the Affymetrix's MAS 5.0 normalization of expression measurements is implemented using the “m*as5*” function with default parameters, and the within-sample normalization is conducted using the “*expresso*” function without cross-sample normalization. Similarly under each of above three pre-processing conditions, the arithmetic mean of all the Gene expression values in each pathway (Mean-G) and their median (Median-G) are also applied in each sample according to previously detailed methods [34]. The resultant pathway boxplots are also presented in **Figure S1** for comparison to those of FAIME.

#### Functional and pathway reproducibility of significantly deregulated FAIME molecular functions and pathways in HNSCC as compared to those of conventional enrichment methods (Figure 3)

Before conducting evaluation of novel approaches, such as diagnosis and prognosis based on sample-level FAIME-derived profiles, we first demonstrate how well FAIME compares with traditional enrichment methods in identifying altered molecular mechanisms between HNSCC tumors and non-tumor control tissues in three independent HNSCC datasets (**Table 1**, datasets A–C). We describe below the overlap among FAIME-derived differentially altered mechanisms, as well as those generated by three conventional approaches: (i) hypergeometric enrichment analysis, (ii) GSEA, and (iii) CORG.

**Three conventional approaches**, hypergeometric enrichment analysis [50], [70], [71], GSEA [18], [23], and CORG [27] are applied to the same three datasets (A,B,C, **Table 1**) to detect KEGG and GO-MF terms (**Text S1**). The KEGG pathway and GO-MF overlap is illustrated as Venn diagrams for each approach (**Figure 3**).

#### *Oncogenic FAIME Features of HNSCC*

FAIME-derived KEGG and GO-MF terms significantly associated with HNSCC malignant transformation are obtained by comparing the 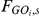 (**Equation 3**) of each KEGG or GO gene-set between the tumor and control tissue groups in each of the three HNSCC datasets (A,B,C, **Table 1**) using a Z-test (paired or unpaired according to the nature of the HNSCC dataset). Before using a Z-test, we also confirm in three HNSCC datasets that FAIME Scores of GO-MFs derived from **Equation 3** meet the same criteria of the normality test as conventional gene profiling (**Figure S2**). To estimate the statistical significance of FAIME-derived mechanisms, a permutation-based test is applied 1000 times for each dataset (resampling sample phenotype assignment), with each application preserves the intra-subcategory relationships of the KEGG and GO terms inherited from the genes. Then, empirical p-values of the observed statistics are calculated from the null distribution using the Bioconductor package *Twilight* [72]. Statistical adjustments for multiple comparisons are done using the Benjamini and Yekutieli method to control for the FDR [73] at ≤5%. The resulting deregulated GO molecular functions are further filtered by the GO-Module approach we developed [74], which removes reported false positive p-values inherited in the GO hierarchy [75], [76] as we previously described [50]. Briefly, GO-Module identifies the representative node for each region that has the smallest adjusted p-value when compared to its graphical contiguous neighbors.

The reproducibility of deregulated FAIME-derived KEGG pathways and GO-MFs between HNSCC tumor samples and non-tumor control tissues is measured by the overlap of the significant mechanisms among the three distinct datasets (***Oncogenic FAIME Features of HNSCC***) as illustrated in the Venn diagrams of **Figure 3**. The statistical significance of these *Oncogenic FAIME Features of HNSCC* is estimated empirically by randomly resampling, without replacement, the same number of predicted KEGG/GO terms from each dataset 1,000 times, and at each time of sampling, the potential 3-way overlap is measured. Additionally, hypergeometric enrichment of GSE6631 expression profile is used as a gold standard (data not shown), in which significantly deregulated FAIME-derived molecular functions and pathways in HNSCC are robustly and consistently present, independent of changing parameters used in analyses such as (i) within array normalization vs. the conventional within and across array normalization (ii) with or without gene-filtering, iii) using a parametric Z test or a non-parametric U-test, and (iv) GO gene annotations downloaded from the NCBI vs. those provided by *Bioconductor GO2ALL*. Furthermore, to demonstrate that mechanism overlap as measured by gene-sets is higher than conventional gene overlap, we compare FAIME-derived GO-MF or KEGG overlap across datasets to the overlap of the original published gene signatures [61]–[63].

#### Methods of evaluation

This section describes four classification evaluations: (a) the concordance of FAIME-derived molecular mechanisms with results obtained by three conventional methods (**Figure 4**), (b–c) the clustering of FAIME-derived profiles of individual samples taken from additional independent datasets for diagnostic sample classification and recurrence-free survival prediction (**Figures 5** **–** **6**), and (d) the validation of recurrence-free survival prediction of FAIME profiles using independent samples.

### Concordance between deregulated FAIME-derived molecular functions and pathways in HNSCC and those obtained by conventional enrichment methods (Figure 4)

In order to objectively assess the accuracy of the significantly deregulated GO-MF and KEGG pathways identified in each of the three HNSCC datasets identified by FAIME (**Figure 3**), a gold standard comprising the true KEGG and GO terms should be used. However, such a gold standard does not exist and published enrichment studies that generated large lists of candidate GO terms and KEGG pathways cannot be thoroughly validated experimentally in their entirety because of the rate limiting nature and cost of such an endeavor. Nonetheless, since a sufficient subset of individual predictions of deregulated molecular functions and pathways from these enrichment studies have been confirmed experimentally, we proceed in using conventional enrichment methods as “proxy-gold standards”. Specifically, GSEA and the hypergeometric enrichments are alternating as proxy-gold standards and as positive controls. These enrichment methods and FAIME are applied to three distinct datasets as described in **Stability and Robustness of FAIME Scores with and without cross-sample normalization and gene filtering** (**Table 1**, datasets A–C). The union of all prioritized GO-MF or KEGG pathways of GSEA or of the hypergeometric analysis illustrated in **Figure 3** is alternatively used as a proxy gold standard. Precision and recall is thus calculated for the FAIME results of a specific dataset and that of the remaining conventional method not used to generate the proxy gold standard. The latter serves as a positive conventional control to compare the accuracy of FAIME-derived results.

#### Evaluation of the utility of *Oncogenic FAIME Features of HNSCC* for diagnostic sample clustering using two additional and independent validation datasets (Figure 5)

The 57 *Oncogenic FAIME Features of HNSCC*, the 33 GO-MFs and 24 KEGG pathways defined in **Figure 3**, serve as seed features to be evaluated in diagnostic validation studies conducted in two independent datasets. To this end, the subset of the Functional FAIME profiles (*FP*; **Equation 4**) comprising these 57 *Oncogenic FAIME Features of HNSCC* (***FP_HNSCC,s_***) are calculated individually for each sample *s* in the validation datasets (**Table 1**: D and E). Then unsupervised hierarchical clustering of the ***FP_HNSCC,s_*** is conducted in each dataset separately as illustrated in **Figure 5**, where the gene-sets of KEGG pathways and GO-MF have at least 3 genes expressed in the independent validation datasets. As we proceed in clustering tumors samples separately from non-tumor control samples we further evaluate whether the accuracy of the results is dependent on the clustering method. The robustness of the *Oncogenic FAIME Features of HNSCC* in separating tumor samples from control tissues is demonstrated by measuring the consistency of the accuracy produced when using different unsupervised clustering approaches (**Table 2**). **Table 2** shows the accuracy of the subcomponents of the *Oncogenic FAIME Features of HNSCC* taken independently, each consisting of GO-MFs or KEGG pathways. The FAIME-Score is used to measure the accuracy and recall of unsupervised sample clustering as compared to their actual clinical condition. In the results section, simulated FAIME-Scores are calculated using the profiles of randomly sampled KEGG/GO terms with the same size as our *Oncogenic FAIME Features of HNSCC* (*n* = 57) to cluster samples in the validation datasets. An empirical p-value is accordingly calculated after running 1000 simulations (**Figure 5**). Additionally, a measure of the area under the curve (AUC) of receiver operator characteristics curve (ROC) is empirically calculated using 100 runs of a five-fold cross-validation [24], [64]. Using the FAIME methods as reported in **Figure S1**, we identified 63 deregulated mechanisms between HNSCC tumors and control tissue with an FDR<5% in both datasets E and F. 39 of these 63 mechanisms recapitulate the 57 *Oncogenic FAIME Features of HNSCC* that are derived at an FDR<5% in datasets A, B and C (*p* = 3×10^−42^, Fisher's Exact Text; n = 2758 mechanisms, FDR<5% in datasets E&F for 63 mechanisms).

#### Proof of concept: utility of FAIME-derived *Oncogenic FAIME Features of HNSCC* for predicting patient survival in two independent validation datasets (Figure 6)

An additional experiment is conducted to confirm that FAIME-derived HNSCC feature*s* are implicated in patient survival. This assumption of the *Oncogenic FAIME Features of HNSCC ’* prognostic power is based on the fact that mechanisms involved in oncogenicity are correlated with patient survival [77], [78]. In order to generate the validation studies of survival shown in **Figure 6**, the subset of the Functional FAIME profiles consisting of the *Oncogenic FAIME Features of HNSCC* (*FP_HNSCC,s_*, FAIME Score Stability, Robustness and Reproducibility Evaluations) are calculated on each tumor sample that has associated patient survival data in the independent datasets E and F separately (**Tables 1** **, S1**). Based on the *FP_HNSCC,s_* of each sample in each dataset, patients are divided into two or three groups using the Bioconductor package *cluster* by the unsupervised partitioning method called “clustering large applications” (CLARA) whose robustness has also been previously shown [79], [80]. Subsequently, Cox proportional hazard regression analyses with censored endpoints are carried out and Kaplan-Meier survival curves are generated by the Bioconductor package *survival* for the predicted clustering of patient samples. The log-rank *p-value* is used to evaluate the prognosis of this sample clustering. Note that in dataset E, duplicate samples are available for 6 patients leading to two *FP_HNSCC,s_*. Thus to avoid duplicate dependent measures for each patient, the average expression of the *FP_HNSCC,s_* is used when partitioning with CLARA.

#### Validated FAIME Scores' ability to reproduce a molecular classification of HNSCC (Figure S6)

Two validation studies are conducted. First, we investigate if these FAIME-derived patterns can recapitulate known biological and pathophysiological knowledge. This is done by comparing the *Oncogenic FAIME Features of HNSCC* we derived with those of a gold standard: the mechanisms that are significantly enriched among genes associated with disease-specific survival [47] (**Figure S6**). We next use FAIME Scores to recapitulate Perou's molecular classification of four “intrinsic” HNSCC sample groups in dataset F [48]. The FAIME-derived profiles are used to stratify HNSCC tumor samples of dataset F in an unsupervised manner into four groups by applying the CLARA algorithm with default parameters on the 57 *Oncogenic FAIME Features of HNSCC*. The R software's Fisher's Exact Test is employed to evaluate the overall enrichment in the resultant 4×4 contingency table consisting of the four FAIME-derived groups of samples and the four HNSCC molecular mechanism groups published by Perou and colleagues.

### FAIME-derived Recurrence-Free Survival (RFS) prognostic mechanisms using Cox proportional hazards (Table S3)

The FAIME-derived mechanism scores of the 208 measurable KEGG pathways and 956 GO-MF terms with more than 3 gene members are calculated for each of the 71 patient samples in dataset E and the 60 patient samples in dataset F. In each dataset, the RFS prognostic power is assessed using a Cox proportional hazards regression with the Bioconductor package *survival* (default parameters for censured data) [81]. In each dataset, a cohort-specific prognostic p-value can thus be calculated for each of these 1,164 mechanisms using the Cox-regression analysis (Bioconductor package *survival*). A meta-analysis of these Cox-regression p-values is then performed using the Stouffer Z-transform method [82] that produces a joint p-value for each mechanism. At each threshold of joint p-values, we obtain a list of prioritized mechanisms for RFS prognosis. Individual FAIME-derived predictors of survival are identified according to a significance threshold of adjusted-p<0.05 after controlling the Stouffer meta-analysis for multiple comparisons using the conservative Bonferroni adjustment (Cox Proportional Hazard applied to datasets E and then F, taking into account the direction of the association for the sign of the Stouffer Z score).

As a validation, the gene members of significant RFS genes are extracted to conduct the principal component analysis. For each mechanism, the resultant 1^st^ component is compared with FAIME Scores for the RFS prognosis analysis. Bioconductor package *amap* is employed to run the PCA analysis and the values of the resultant 1st component are compared with FAIME Scores for each patient in dataset E and F. Two types of analyses are conducted: 1) the non-parametric Spearman correlation is calculated between 1^st^ component values and FAIME Scores across all samples, and 2) the Cox regression analyses of RFS are conducted for both individual FAIME Scores and 1st component values for each sample. Note that for the patient with duplicate measured samples in dataset E, we use the mean expression value to represent its expression measurement and genes with non-measurable expression values are assigned to a value of zero in Dataset F.

## Supporting Information

Figure S1 FAIME pathway scores are more stable under three preprocessing conditions than those of conventional pathway scoring (Mean-G, Median-G).
(EPS)

Figure S2 The normality of FAIME-profiles are comparable to the normality of gene expression.
(EPS)

Figure S3 The accuracy of differentially expressed FAIME profiles using pooled GSEA results as a “proxy gold standard”.
(EPS)

Figure S4 Significantly altered *Oncogenic FAIME Features of HNSCC* accurately classify samples of an independent dataset of laser microdissected HNSCC tumors (GSE9844).
(EPS)

Figure S5 Oncogenic FAIME Features of HNSCC stratify recurrence-free survival in independent dataset GSE2837.
(EPS)

Figure S6 Enrichment between *Oncogenic FAIME Features of HNSCC* and FA-gene enriched mechanisms.
(EPS)

Figure S7 FAIME Score of the KEGG apoptosis geneset is significantly increased in patients with no evidence of disease (NED) recurrence.
(EPS)

Table S1 57 *Oncogenic FAIME Features of HNSCC* identified between tumor tissue and non-tumor control tissue in all three HNSCC datasets (A,B,C).
(PDF)

Table S2 Gene-based vs. FAIME-based classification of HNSCC tumor vs. non-tumor control samples.
(PDF)

Table S3 Two Prioritized Recurrence-Free Survival (RFS) prognostic mechanisms identified by FAIME in two HNSCC datasets (E,F).
(PDF)

Table S4 KEGG apoptosis geneset (hsa04210) measurable among 26 laser microdissected head and neck tumor samples (GSE9844).
(PDF)

Table S5 Head and neck tumor samples.
(PDF)

Table S6 47 significant Oncogenic FAIME Features of HNSCC confirmed in laser microdissected HNSCC dataset GSE9844.
(PDF)

Table S7 Non-tumor control samples.
(PDF)

Table S8 Clinical features.
(PDF)

Text S1 Implementation of Enrichment, GSEA and CORG.
(PDF)

Text S2 R codes to run FAIME.
(PDF)

## References

[1] van de Vijver MJ, He YD, van't Veer LJ, Dai H, Hart AA (2002). A gene-expression signature as a predictor of survival in breast cancer.. N Engl J Med.

[2] Paik S, Shak S, Tang G, Kim C, Baker J (2004). A multigene assay to predict recurrence of tamoxifen-treated, node-negative breast cancer.. N Engl J Med.

[3] Chuang HY, Lee E, Liu YT, Lee D, Ideker T (2007). Network-based classification of breast cancer metastasis.. Mol Syst Biol.

[4] Minn AJ, Gupta GP, Siegel PM, Bos PD, Shu W (2005). Genes that mediate breast cancer metastasis to lung.. Nature.

[5] Ogawa K, Murayama S, Mori M (2007). Predicting the tumor response to radiotherapy using microarray analysis (Review).. Oncol Rep.

[6] Friedman DR, Weinberg JB, Barry WT, Goodman BK, Volkheimer AD (2009). A genomic approach to improve prognosis and predict therapeutic response in chronic lymphocytic leukemia.. Clin Cancer Res.

[7] Fan C, Oh DS, Wessels L, Weigelt B, Nuyten DS (2006). Concordance among gene-expression-based predictors for breast cancer.. N Engl J Med.

[8] Chen J, Sam L, Huang Y, Lee Y, Li J (2010). Protein interaction network underpins concordant prognosis among heterogeneous breast cancer signatures.. J Biomed Inform.

[9] Massague J (2007). Sorting out breast-cancer gene signatures.. N Engl J Med.

[10] Ogata H, Goto S, Sato K, Fujibuchi W, Bono H (1999). KEGG: Kyoto Encyclopedia of Genes and Genomes.. Nucleic Acids Res.

[11] Ashburner M, Ball CA, Blake JA, Botstein D, Butler H (2000). Gene ontology: tool for the unification of biology. The Gene Ontology Consortium.. Nat Genet.

[12] Gong X, Wu R, Zhang Y, Zhao W, Cheng L (2010). Extracting consistent knowledge from highly inconsistent cancer gene data sources.. BMC Bioinformatics.

[13] van Vliet MH, Reyal F, Horlings HM, van de Vijver MJ, Reinders MJ (2008). Pooling breast cancer datasets has a synergetic effect on classification performance and improves signature stability.. BMC Genomics.

[14] Huang da W, Sherman BT, Tan Q, Collins JR, Alvord WG (2007). The DAVID Gene Functional Classification Tool: a novel biological module-centric algorithm to functionally analyze large gene lists.. Genome Biol.

[15] Beissbarth T, Speed TP (2004). GOstat: find statistically overrepresented Gene Ontologies within a group of genes.. Bioinformatics.

[16] Henegar C, Cancello R, Rome S, Vidal H, Clement K (2006). Clustering biological annotations and gene expression data to identify putatively co-regulated biological processes.. J Bioinform Comput Biol.

[17] Henegar C, Tordjman J, Achard V, Lacasa D, Cremer I (2008). Adipose tissue transcriptomic signature highlights the pathological relevance of extracellular matrix in human obesity.. Genome Biol.

[18] Subramanian A, Tamayo P, Mootha VK, Mukherjee S, Ebert BL (2005). Gene set enrichment analysis: a knowledge-based approach for interpreting genome-wide expression profiles.. Proc Natl Acad Sci U S A.

[19] Bauer S, Gagneur J, Robinson PN (2010). GOing Bayesian: model-based gene set analysis of genome-scale data.. Nucleic Acids Res.

[20] Engreitz JM, Daigle BJ, Marshall JJ, Altman RB (2010). Independent component analysis: mining microarray data for fundamental human gene expression modules.. J Biomed Inform.

[21] Al-Shahrour F, Arbiza L, Dopazo H, Huerta-Cepas J, Minguez P (2007). From genes to functional classes in the study of biological systems.. BMC Bioinformatics.

[22] Goeman JJ, van de Geer SA, de Kort F, van Houwelingen HC (2004). A global test for groups of genes: testing association with a clinical outcome.. Bioinformatics.

[23] Nam D, Kim SY (2008). Gene-set approach for expression pattern analysis.. Brief Bioinform.

[24] Dinu I, Potter JD, Mueller T, Liu Q, Adewale AJ (2009). Gene-set analysis and reduction.. Brief Bioinform.

[25] Ackermann M, Strimmer K (2009). A general modular framework for gene set enrichment analysis.. BMC Bioinformatics.

[26] Yang D, Li Y, Xiao H, Liu Q, Zhang M (2008). Gaining confidence in biological interpretation of the microarray data: the functional consistence of the significant GO categories.. Bioinformatics.

[27] Lee E, Chuang HY, Kim JW, Ideker T, Lee D (2008). Inferring pathway activity toward precise disease classification.. PLoS Comput Biol.

[28] Abraham G, Kowalczyk A, Loi S, Haviv I, Zobel J (2010). Prediction of breast cancer prognosis using gene set statistics provides signature stability and biological context.. BMC Bioinformatics.

[29] Ma S, Kosorok MR (2010). Detection of gene pathways with predictive power for breast cancer prognosis.. BMC Bioinformatics.

[30] Watters JW, Roberts CJ (2006). Developing gene expression signatures of pathway deregulation in tumors.. Mol Cancer Ther.

[31] Gatza ML, Lucas JE, Barry WT, Kim JW, Wang Q (2010). A pathway-based classification of human breast cancer.. Proc Natl Acad Sci U S A.

[32] Acharya CR, Hsu DS, Anders CK, Anguiano A, Salter KH (2008). Gene expression signatures, clinicopathological features, and individualized therapy in breast cancer.. JAMA.

[33] He Z, Yu W (2010). Stable feature selection for biomarker discovery.. Comput Biol Chem.

[34] Guo Z, Zhang T, Li X, Wang Q, Xu J (2005). Towards precise classification of cancers based on robust gene functional expression profiles.. BMC Bioinformatics.

[35] Bild AH, Yao G, Chang JT, Wang Q, Potti A (2006). Oncogenic pathway signatures in human cancers as a guide to targeted therapies.. Nature.

[36] Tomfohr J, Lu J, Kepler TB (2005). Pathway level analysis of gene expression using singular value decomposition.. BMC Bioinformatics.

[37] Chen X, Wang L (2009). Integrating biological knowledge with gene expression profiles for survival prediction of cancer.. J Comput Biol.

[38] Su J, Yoon BJ, Dougherty ER (2009). Accurate and reliable cancer classification based on probabilistic inference of pathway activity.. PLoS One.

[39] Seiwert TY, Cohen EE (2005). State-of-the-art management of locally advanced head and neck cancer.. Br J Cancer.

[40] Edge SB, Byrd DR, Compton CC, Fritz AG, Greene FL (2009). AJCC Cancer Staging Manual. 7th edition.

[41] Mouw KW, Haraf DJ, Stenson KM, Cohen EE, Xi X (2010). Factors associated with long-term speech and swallowing outcomes after chemoradiotherapy for locoregionally advanced head and neck cancer.. Arch Otolaryngol Head Neck Surg.

[42] Cohen EE, Haraf DJ, Kunnavakkam R, Stenson KM, Blair EA (2010). Epidermal growth factor receptor inhibitor gefitinib added to chemoradiotherapy in locally advanced head and neck cancer.. J Clin Oncol.

[43] Sontrop HM, Moerland PD, van den Ham R, Reinders MJ, Verhaegh WF (2009). A comprehensive sensitivity analysis of microarray breast cancer classification under feature variability.. BMC Bioinformatics.

[44] Vander Heiden MG, Cantley LC, Thompson CB (2009). Understanding the Warburg effect: the metabolic requirements of cell proliferation.. Science.

[45] Jones RG, Thompson CB (2009). Tumor suppressors and cell metabolism: a recipe for cancer growth.. Genes Dev.

[46] Pyeon D, Newton MA, Lambert PF (2007). Fundamental differences in cell cycle deregulation in human papillomavirus-positive and human papillomavirus-negative head/neck and cervical cancers.. Cancer Res.

[47] Thurlow JK, Pena Murillo CL, Hunter KD, Buffa FM, Patiar S (2010). Spectral clustering of microarray data elucidates the roles of microenvironment remodeling and immune responses in survival of head and neck squamous cell carcinoma.. J Clin Oncol.

[48] Chung CH, Parker JS, Karaca G, Wu J, Funkhouser WK (2004). Molecular classification of head and neck squamous cell carcinomas using patterns of gene expression.. Cancer Cell.

[49] Yang X, Bentink S, Scheid S, Spang R (2006). Similarities of ordered gene lists.. J Bioinform Comput Biol.

[50] Lee Y, Yang X, Huang Y, Fan H, Zhang Q (2010). Network Modeling Identifies Molecular Functions Targeted by miR-204 to Suppress Head and Neck Tumor Metastasis.. PLoS Comput Biol.

[51] Zilliox MJ, Irizarry RA (2007). A gene expression bar code for microarray data.. Nature methods.

[52] McCall MN, Uppal K, Jaffee HA, Zilliox MJ, Irizarry RA (2011). The Gene Expression Barcode: leveraging public data repositories to begin cataloging the human and murine transcriptomes.. Nucleic Acids Res.

[53] Liu Y, Sam L, Li J, Lussier YA (2009). Robust methods for accurate diagnosis using pan-microbiological oligonucleotide microarrays.. BMC Bioinformatics.

[54] Malanoski AP, Lin B, Wang Z, Schnur JM, Stenger DA (2006). Automated identification of multiple micro-organisms from resequencing DNA microarrays.. Nucleic Acids Res.

[55] Palacios G, Quan PL, Jabado OJ, Conlan S, Hirschberg DL (2007). Panmicrobial oligonucleotide array for diagnosis of infectious diseases.. Emerg Infect Dis.

[56] Chiu CY, Alizadeh AA, Rouskin S, Merker JD, Yeh E (2007). Diagnosis of a critical respiratory illness caused by human metapneumovirus by use of a pan-virus microarray.. J Clin Microbiol.

[57] Slaughter DP, Southwick HW, Smejkal W (1953). Field cancerization in oral stratified squamous epithelium; clinical implications of multicentric origin.. Cancer.

[58] Huang E, Ishida S, Pittman J, Dressman H, Bild A (2003). Gene expression phenotypic models that predict the activity of oncogenic pathways.. Nat Genet.

[59] Knauer M, Mook S, Rutgers EJ (2010). The predictive value of the 70-gene signature for adjuvant chemotherapy in early breast cancer.. Breast Cancer Res Treat.

[60] Massagué J (2007). Sorting out breast-cancer signatures.. N Engl J Med.

[61] Kuriakose MA, Chen WT, He ZM, Sikora AG, Zhang P (2004). Selection and validation of differentially expressed genes in head and neck cancer.. Cell Mol Life Sci.

[62] Cromer A, Carles A, Millon R, Ganguli G, Chalmel F (2004). Identification of genes associated with tumorigenesis and metastatic potential of hypopharyngeal cancer by microarray analysis.. Oncogene.

[63] Colella S, Richards KL, Bachinski LL, Baggerly KA, Tsavachidis S (2008). Molecular signatures of metastasis in head and neck cancer.. Head Neck.

[64] Ye H, Yu T, Temam S, Ziober BL, Wang J (2008). Transcriptomic dissection of tongue squamous cell carcinoma.. BMC Genomics.

[65] Chung CH, Parker JS, Ely K, Carter J (2006). Gene expression profiles identify epithelial-to-mesenchymal transition and activation of nuclear factor-kappaB signaling as characteristics of a high-risk head and neck squamous cell carcinoma.. Cancer Res.

[66] Falcon S, Gentleman R (2008). Hypergeometric Testing Used for Gene Set Enrichment Analysis. Bioconductor Case Studies.

[67] Lottaz C, Yang X, Scheid S, Spang R (2006). OrderedList–a bioconductor package for detecting similarity in ordered gene lists.. Bioinformatics.

[68] Affymetrix (2002). GeneChip® Expression Analysis: Data Analysis Fundamentals..

[69] Gentleman RC, Carey VJ, Bates DM, Bolstad B, Dettling M (2004). Bioconductor: open software development for computational biology and bioinformatics.. Genome Biol.

[70] Grossmann S, Bauer S, Robinson PN, Vingron M (2006). An Improved Statistic for Detecting Over-Represented Gene Ontology Annotations in Gene Sets. Research in Computational Molecular Biology.

[71] Falcon S, Gentleman R (2007). Using GOstats to test gene lists for GO term association.. Bioinformatics.

[72] Scheid S, Spang R (2005). twilight; a Bioconductor package for estimating the local false discovery rate.. Bioinformatics.

[73] Benjamini Y, Yekutieli D (2001). The control of the false discovery rate in multiple testing under dependency.. Ann Statist.

[74] Yang X, Li J, Lee Y, Lussier YA (2011). GO-Module: functional synthesis and improved interpretation of Gene Ontology patterns.. Bioinformatics.

[75] Barry WT, Nobel AB, Wright FA (2005). Significance analysis of functional categories in gene expression studies: a structured permutation approach.. Bioinformatics.

[76] Prufer K, Muetzel B, Do HH, Weiss G, Khaitovich P (2007). FUNC: a package for detecting significant associations between gene sets and ontological annotations.. BMC Bioinformatics.

[77] Horowitz J (1999). Adenovirus-mediated p53 gene therapy: overview of preclinical studies and potential clinical applications.. Curr Opin Mol Ther.

[78] Giri U, Ashorn CL, Ramdas L, Stivers DN, Coombes K (2006). Molecular signatures associated with clinical outcome in patients with high-risk head-and-neck squamous cell carcinoma treated by surgery and radiation.. Int J Radiat Oncol Biol Phys.

[79] Kaufman LR, P. J (1990). Finding Groups in Data: An Introduction to Cluster Analysis.

[80] Bozinov D, Rahnenfuhrer J (2002). Unsupervised technique for robust target separation and analysis of DNA microarray spots through adaptive pixel clustering.. Bioinformatics.

[81] Andersen PK, Gill RD (1982). Cox's Regression Model for Counting Processes: A Large Sample Study.. Ann Statist.

[82] Whitlock MC (2005). Combining probability from independent tests: the weighted Z-method is superior to Fisher's approach.. J Evol Biol.

