# Correction: Single Sample Expression-Anchored Mechanisms Predict Survival in Head and Neck Cancer

**DOI:** 10.1371/journal.pcbi.1003609

**Published:** 2014-05-02

**Authors:** The PLOS Computational Biology Staff

There is an error in the Methods section, subsection ‘FAIME profile’, second paragraph. The correct sentence is: To quantitatively assign a mechanism's “expression deregulation” via its gene members, whose expression is measured in a microarray, all expressed genes (set ***G***) in each sample are sorted in an ascending order according to their expression levels, and then an exponential decreasing weight (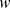) is assigned to the ordered genes (**Equation 1**).

In addition, there is an error in Equation 1 in the Methods section. The sign of the exponent of the exponential function should be removed. Please view the complete, correct equation here:
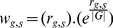
(1)

