# Supplementary material for: Single Sample Expression-Anchored Mechanisms Predict Survival in Head and Neck Cancer
Source: PLoS Comput Biol. 2012 Jan 26;8(1):e1002350. doi: 10.1371/journal.pcbi.1002350 (PMC3266878; doi:10.1371/journal.pcbi.1002350)
Supplement: Table S1 — 57 Oncogenic FAIME Features of HNSCC identified between tumor tissue and non-tumor control tissue in all three HNSCC datasets (A,B,C). (PDF) [file pcbi.1002350.s008.pdf]

Table S1. 57 HNSCC FAIME Features identified between tumor tissue and non-tumor control tissue in all three HNSCC datasets. References are provided for mechanisms that were also found by other approaches (e.g. enrichment, CORG, and/or GSEA) comparing tumor to normal tissues (**Figure 3**), and for co-expressed clusters found in **Figure 5**.

**HNSCC FAIME Features**	**up- or down-regulated in HNSCC tumor samples** (FAIME score, **Methods, Fig. 3,5)**	**Confirmed by enrichment, GSEA or CORG** (**Fig. 3**; # of methods; found in any of datasets A-C)	**Scale**	**ID**	**# of genes** (GO from NCBI on Dec. 11, 09; Bioconductor *kegg.db* v2.3.5 on Sep 16, 09)	**Cluster (Fig. 5)**
Bladder cancer	↑	**3**	KEGG	hsa05219	42	**α**
ECM-receptor interaction	↑	**3**	KEGG	hsa04512	84	**α**
extracellular matrix binding	↑	**3**	GO-MF	GO:0050840	13	**α**
Focal adhesion	↑	**3**	KEGG	hsa04510	202	**α**
glucuronosyl-N-acetylgalactosaminyl-proteoglycan 4-beta-N-acetylgalactosaminyltransferase activity	↑	**1**	GO-MF	GO:0047238	4	**α**
Glycosaminoglycan biosynthesis - chondroitin sulfate	↑	**0**	KEGG	hsa00532	22	**α**
L-ascorbic acid binding	↑	**3**	GO-MF	GO:0031418	21	**α**
metalloendopeptidase activity	↑	**3**	GO-MF	GO:0004222	104	**α**
N-acetylgalactosaminyl-proteoglycan 3-beta-glucuronosyltransferase activity	↑	**1**	GO-MF	GO:0050510	4	**α**
oxidoreductase activity, acting on paired donors, with incorporation or reduction of molecular oxygen	↑	**2**	GO-MF	GO:0016705	16	**α**
p53 signaling pathway	↑	**3**	KEGG	hsa04115	69	**α**
Pathways in cancer	↑	**3**	KEGG	hsa05200	331	**α**
platelet-derived growth factor binding	↑	**2**	GO-MF	GO:0048407	11	**α**
procollagen-proline 4-dioxygenase activity	↑	**1**	GO-MF	GO:0004656	4	**α**
Small cell lung cancer	↑	**3**	KEGG	hsa05222	86	**α**
Base excision repair	↑	**1**	KEGG	hsa03410	35	**β**
Cell cycle	↑	**3**	KEGG	hsa04110	129	**β**
cyclin-dependent protein kinase regulator activity	↑	**1**	GO-MF	GO:0016538	6	**β**
DNA replication	↑	**2**	KEGG	hsa03030	36	**β**
Mismatch repair	↑	**0**	KEGG	hsa03430	23	**β**
Pyrimidine metabolism	↑	**2**	KEGG	hsa00240	97	**β**
ribonuclease H activity	↑	**1**	GO-MF	GO:0004523	7	**β**
metabolism	↓	**2**	KEGG	hsa00650	34	**γ**
1- and 2-Methylnaphthalene degradation	↓	**2**	KEGG	hsa00624	13	**γ**
3-Chloroacrylic acid degradation	↓	**2**	KEGG	hsa00641	12	**γ**
3-chloroallyl aldehyde dehydrogenase activity	↓	**1**	GO-MF	GO:0004028	4	**γ**

acyl-CoA dehydrogenase activity	↓	**1**	GO-MF	GO:0003995	9	γ
acyl-CoA oxidase activity	↓	**0**	GO-MF	GO:0003997	4	γ
acylglycerol lipase activity	↓	**0**	GO-MF	GO:0047372	6	γ
alcohol dehydrogenase activity, zinc-dependent	↓	**2**	GO-MF	GO:0004024	4	γ
aldehyde dehydrogenase (NAD) activity	↓	**2**	GO-MF	GO:0004029	8	γ
aldehyde dehydrogenase [NAD(P)+] activity	↓	**2**	GO-MF	GO:0004030	6	γ
Arachidonic acid metabolism	↓	**3**	KEGG	hsa00590	58	γ
aromatase activity	↓	**2**	GO-MF	GO:0070330	24	γ
Ascorbate and aldarate metabolism	↓	**1**	KEGG	hsa00053	25	γ
beta-Alanine metabolism	↓	**2**	KEGG	hsa00410	22	γ
Drug metabolism - cytochrome P450	↓	**3**	KEGG	hsa00982	72	γ
epoxide hydrolase activity	↓	**1**	GO-MF	GO:0004301	4	γ
Fatty acid metabolism	↓	**2**	KEGG	hsa00071	42	γ
Glycerolipid metabolism	↓	**2**	KEGG	hsa00561	45	γ
Glycolysis / Gluconeogenesis	↓	**2**	KEGG	hsa00010	62	γ
Histidine metabolism	↓	**2**	KEGG	hsa00340	29	γ
Linoleic acid metabolism	↓	**2**	KEGG	hsa00591	30	γ
Metabolism of xenobiotics by cytochrome P450	↓	**3**	KEGG	hsa00980	70	γ
Phenylalanine metabolism	↓	**1**	KEGG	hsa00360	21	γ
polysaccharide binding	↓	**1**	GO-MF	GO:0030247	11	γ
Primary bile acid biosynthesis	↓	**1**	KEGG	hsa00120	16	γ
Propanoate metabolism	↓	**2**	KEGG	hsa00640	33	γ
protein tyrosine kinase activator activity	↓	**1**	GO-MF	GO:0030296	4	γ
protein-glutamine gamma-glutamyltransferase activity	↓	**2**	GO-MF	GO:0003810	9	γ
Retinol metabolism	↓	**2**	KEGG	hsa00830	64	γ
serine-type peptidase activity	↓	**1**	GO-MF	GO:0008236	22	γ
structural constituent of myelin sheath	↓	**2**	GO-MF	GO:0019911	4	γ
Sulfur metabolism	↓	**1**	KEGG	hsa00920	13	γ
Tropane, piperidine and pyridine alkaloid biosynthesis	↓	**2**	KEGG	hsa00960	9	γ
Tyrosine metabolism	↓	**2**	KEGG	hsa00350	47	γ
Valine, leucine and isoleucine degradation	↓	**2**	KEGG	hsa00280	44	γ
