# Supplementary material for: Single Sample Expression-Anchored Mechanisms Predict Survival in Head and Neck Cancer
Source: PLoS Comput Biol. 2012 Jan 26;8(1):e1002350. doi: 10.1371/journal.pcbi.1002350 (PMC3266878; doi:10.1371/journal.pcbi.1002350)
Supplement: Table S2 — Gene-based vs. FAIME-based classification of HNSCC tumor vs. non-tumor control samples. (PDF) [file pcbi.1002350.s009.pdf]

Table S2: Gene-based vs. FAIME-based classification of HNSCC tumor vs. non-tumor control samples. *Oncogenic FAIME Features of HNSCC* (**Methods**) were derived from datasets A, B and C (**Figure 3**), and tested in datasets D and E for its ability to discriminate between tumor vs. non-tumor control tissue. As shown below, FAIME-based classification required less features than Gene-based classification, and obtained equivalent or better accuracy scores. Note that the accuracies reported in the top rows under "Gene-based analysis" are extracted from the original mansucripts cited in the Reference row.

**Dataset ID**	**A**	**B**	**C**	**D**	**E**
**Reference**	GSE6631 [61]	GSE2379 [62]	E-MEXP-44 [63]	E-MEXP-44 [63]	JCO2010 [47]
**Gene-based analysis**					
Computational method	H-clustering	PAM; cross-validation	PCA; H-clustering	PCA; H-clustering	Spectral clustering
# probe features	42	121	338	338	1000
Accuracy for tumor	100%	94%	100%	100%	99%
Accuracy for non-tumor control	59%	100%	74%	74%	86%
Overall Accuracy	71%	95%	90%	90%	96%
**FAIME analysis**					
# mechanism features	**Training sets**			**57**	**57**
Accuracy for tumor				**100%**	**97%**
Accuracy for non-tumor control				**100%**	**86%**
Overall Accuracy				**100%**	**96%**

Legend: PAM: Partitioning Around Medoids clustering; PCA: Principal component analysis; H-clustering: Hierarchical clustering.
