# Supplementary material for: Single Sample Expression-Anchored Mechanisms Predict Survival in Head and Neck Cancer
Source: PLoS Comput Biol. 2012 Jan 26;8(1):e1002350. doi: 10.1371/journal.pcbi.1002350 (PMC3266878; doi:10.1371/journal.pcbi.1002350)
Supplement: Table S3 — Two Prioritized Recurrence-Free Survival (RFS) prognostic mechanisms identified by FAIME in two HNSCC datasets (E,F). (PDF) [file pcbi.1002350.s010.pdf]

Table S3. Five prioritized Recurrence-Free Survival prognostic mechanisms identified by FAIME in two HNSCC datasets (E,F). FAIME scores of each of the 208 measurable KEGG pathways and 956 GO-MF mechanism is first calculated in each tumor sample. The Cox proportional hazard is calculated for each mechanism in dataset E using the time to recurrence associated with each sample and then repeated in dataset F producing the unadjusted "prognostic p-values" shown in the table (**Methods**). Finally, a "joint prognostic p-value" was calculated from these two individual dataset prognostic p-values for each of these mechanisms using Stouffer meta-analysis. Presented here are mechanisms with Bonferroni adjustment joint p-values <0.05 thus controlled for multiple comparions. A Spearman non-parametric correlation was also calculated between the FAIME scores and the 1st component of Principle Component Analysis (PCA) values across samples in each dataset.

**Recurrence-Free Survival (RFS) prognostic mechanisms predictors**	**ID**	**# genes**	**Cox Proprotional Hazards of Recurrence-Free Survival**						**Spearman Correlation of FAIME-Scores with those of the 1st Component of the Principal Component Analysis**	
			**P-values**				**Cox Regression Coefficients***			
			unadjusted P in Dataset E	unadjusted P in Dataset F	unadjusted Stouffer meta-analysis Pvalue	**Bonferroni-adjusted Stouffer meta-analysis**	**Dataset E**	**Dataset F**	Pvalue in Dataset E	Pvalue in Dataset F
Apoptosis	hsa04210	89	2.38E-04	5.91E-04	9.51E-07	**2.62E-03**	5.91E-04	4.69E-02	9.51E-07	2.62E-03
receptor signaling complex scaffold activity	GO:0030159	13	2.52E-07	5.04E-02	1.22E-06	**3.35E-03**	5.04E-02	3.89E-02	1.22E-06	3.35E-03

*a negative coefficient indicates is associated with a higher FAIME score in a sample

**Recurrence-Free Survival (RFS) prognostic mechanisms predictors**	**ID**	**# genes**	**Dataset E**		**Dataset F**		**Stouffer meta-analysis**	
			**Cox regression test P**	**Cox regression coefficient***	**Cox regression test P**	**Cox regression coefficient***	**unadjusted p**	**Bonferroni adjusted p**
Apoptosis	hsa04210	89	2.38E-04	-3.42E-03	5.91E-04	-5.44E-03	9.51E-07	2.62E-03
receptor signaling complex scaffold activity	GO:0030159	13	2.52E-07	-1.64E-03	5.04E-02	-9.55E-04	1.22E-06	3.35E-03

*a positive coefficient indicates the higher a FAIME score, the poorer the prognosis; a negative coefficient indicates the higher a FAIME score, the better the prognosis.
