# Supplementary material for: Single Sample Expression-Anchored Mechanisms Predict Survival in Head and Neck Cancer
Source: PLoS Comput Biol. 2012 Jan 26;8(1):e1002350. doi: 10.1371/journal.pcbi.1002350 (PMC3266878; doi:10.1371/journal.pcbi.1002350)
Supplement: Table S4 — KEGG apoptosis geneset (hsa04210) measurable among 26 laser microdissected head and neck tumor samples (GSE9844). (PDF) [file pcbi.1002350.s011.pdf]

Table S4. KEGG apoptosis geneset (hsa04210) measurable among 26 laser microdissected head and neck tumor samples (GSE9844). The average expression values (log2 scale) of gene members in the KEGG apoptosis geneset are reported for HNSCC laser microdissected dataset GSE9844.

Gene Symbol	Gene Description	Average expression
PIK3CB	phosphoinositide-3-kinase, catalytic, beta polypeptide	5.03
TP53	tumor protein p53	3.72
BIRC3	baculoviral IAP repeat-containing 3	4.72
CSF2RB	colony stimulating factor 2 receptor, beta, low-affinity (granulocyte-macrophage)	6.97
AKT3	v-akt murine thymoma viral oncogene homolog 3 (protein kinase B, gamma)	4.24
PRKAR1B	protein kinase, cAMP-dependent, regulatory, type I, beta	5.58
PIK3CG	phosphoinositide-3-kinase, catalytic, gamma polypeptide	3.04
PIK3R3	phosphoinositide-3-kinase, regulatory subunit 3 (gamma)	3.82
PIK3R2	phosphoinositide-3-kinase, regulatory subunit 2 (beta)	5.60
IKBKB	inhibitor of kappa light polypeptide gene enhancer in B-cells, kinase beta	5.03
CASP8	caspase 8, apoptosis-related cysteine peptidase	4.32
CASP10	caspase 10, apoptosis-related cysteine peptidase	4.16
DFFA	DNA fragmentation factor, 45kDa, alpha polypeptide	5.81
TNFRSF10C	tumor necrosis factor receptor superfamily, member 10c, decoy without an intracellular domain	3.92
CHP	calcium binding protein P22	6.42
BCL2L1	BCL2-like 1	4.40
PRKAR2A	protein kinase, cAMP-dependent, regulatory, type II, alpha	7.15
IRAK4	interleukin-1 receptor-associated kinase 4	3.58
CASP3	caspase 3, apoptosis-related cysteine peptidase	4.16
IRAK2	interleukin-1 receptor-associated kinase 2	4.34
CHUK	conserved helix-loop-helix ubiquitous kinase	4.87
CFLAR	CASP8 and FADD-like apoptosis regulator	5.07
CYCS	cytochrome c, somatic	6.01
IRAK1	interleukin-1 receptor-associated kinase 1	7.56
PPP3CB	protein phosphatase 3, catalytic subunit, beta isozyme	6.75
BAD	BCL2-associated agonist of cell death	3.75
IL1R1	interleukin 1 receptor, type I	5.72
IL1A	interleukin 1, alpha	4.37
PIK3CA	phosphoinositide-3-kinase, catalytic, alpha polypeptide	3.96
IL1B	interleukin 1, beta	6.90
PPP3CC	protein phosphatase 3, catalytic subunit, gamma isozyme	3.88
ATM	ataxia telangiectasia mutated	3.60
PRKAR2B	protein kinase, cAMP-dependent, regulatory, type II, beta	3.59

TNFRSF10D	tumor necrosis factor receptor superfamily, member 10d, decoy with truncated death domain	4.82
TNF	tumor necrosis factor	4.23
FASLG	Fas ligand (TNF superfamily, member 6)	4.00
IRAK3	interleukin-1 receptor-associated kinase 3	3.22
PIK3CD	phosphoinositide-3-kinase, catalytic, delta polypeptide	5.15
CASP9	caspase 9, apoptosis-related cysteine peptidase	4.64
PRKAR1A	protein kinase, cAMP-dependent, regulatory, type I, alpha (tissue specific extinguisher 1)	6.26
MYD88	myeloid differentiation primary response gene (88)	8.02
BCL2	B-cell CLL/lymphoma 2	4.35
PPP3CA	protein phosphatase 3, catalytic subunit, alpha isozyme	7.17
PRKX	protein kinase, X-linked	4.15
APAF1	apoptotic peptidase activating factor 1	3.89
TNFSF10	tumor necrosis factor (ligand) superfamily, member 10	8.00
IL1RAP	interleukin 1 receptor accessory protein	4.37
PIK3R1	phosphoinositide-3-kinase, regulatory subunit 1 (alpha)	4.80
CAPN2	calpain 2, (m/II) large subunit	8.03
CASP7	caspase 7, apoptosis-related cysteine peptidase	7.00
CASP6	caspase 6, apoptosis-related cysteine peptidase	4.75
FADD	Fas (TNFRSF6)-associated via death domain	6.01
PRKACB	protein kinase, cAMP-dependent, catalytic, beta	4.95
RIPK1	receptor (TNFRSF)-interacting serine-threonine kinase 1	4.44
MAP3K14	mitogen-activated protein kinase kinase kinase 14	4.15
PRKACA	protein kinase, cAMP-dependent, catalytic, alpha	5.31
PRKY	protein kinase, Y-linked	4.05
NTRK1	neurotrophic tyrosine kinase, receptor, type 1	3.00
XIAP	X-linked inhibitor of apoptosis	5.02
BID	BH3 interacting domain death agonist	7.04
AIFM1	apoptosis-inducing factor, mitochondrion-associated, 1	6.38
CAPN1	calpain 1, (mu/I) large subunit	5.76
BAX	BCL2-associated X protein	3.97
CHP2	calcineurin B homologous protein 2	4.60
DFFB	DNA fragmentation factor, 40kDa, beta polypeptide (caspase-activated DNase)	5.69
AKT2	v-akt murine thymoma viral oncogene homolog 2	4.77
AKT1	v-akt murine thymoma viral oncogene homolog 1	3.47
IL3	interleukin 3 (colony-stimulating factor, multiple)	3.00
TNFRSF10B	tumor necrosis factor receptor superfamily, member 10b	6.43
TNFRSF1A	tumor necrosis factor receptor superfamily, member 1A	7.93
PPP3R1	protein phosphatase 3, regulatory subunit B, alpha	3.85
ENDOG	endonuclease G	5.87
NFKBIA	nuclear factor of kappa light polypeptide gene enhancer in B-cells inhibitor, alpha	6.69

RELA	v-rel reticuloendotheliosis viral oncogene homolog A (avian)	4.95
FAS	Fas (TNF receptor superfamily, member 6)	3.94
BIRC2	baculoviral IAP repeat-containing 2	9.85
IL3RA	interleukin 3 receptor, alpha (low affinity)	4.98
NFKB1	nuclear factor of kappa light polypeptide gene enhancer in B-cells 1	4.71
TRAF2	TNF receptor-associated factor 2	4.98
IKBKG	inhibitor of kappa light polypeptide gene enhancer in B-cells, kinase gamma	6.22
TNFRSF10A	tumor necrosis factor receptor superfamily, member 10a	4.16
EXOG	endo/exonuclease (5'-3'), endonuclease G-like	3.81
ENDOD1	endonuclease domain containing 1	6.43
PIK3R5	phosphoinositide-3-kinase, regulatory subunit 5	3.96
NGF	nerve growth factor (beta polypeptide)	4.46
PPP3R2	protein phosphatase 3, regulatory subunit B, beta	3.26
PRKACG	protein kinase, cAMP-dependent, catalytic, gamma	4.15
TRADD	TNFRSF1A-associated via death domain	5.61
LOC651610	similar to Serine-protein kinase ATM (Ataxia telangiectasia mutated) (A-T, mutated)	N/A
