# Supplementary material for: Single Sample Expression-Anchored Mechanisms Predict Survival in Head and Neck Cancer
Source: PLoS Comput Biol. 2012 Jan 26;8(1):e1002350. doi: 10.1371/journal.pcbi.1002350 (PMC3266878; doi:10.1371/journal.pcbi.1002350)
Supplement: Table S5 — Head and neck tumor samples. (PDF) [file pcbi.1002350.s012.pdf]

Table S5. Head and Neck Tumor Samples. Shown below are the details on the total number of tumors, tumor location, tumor cell composition, and method of extraction for each dataset in our study as reported in the original publications cited in the Reference column.

**ID in the manuscript**	**Reference**	**Total # of Tumors**	**Tumor location**: OC=oral cavity, OP=oropharynx, HP=Hypopharynx, L=larynx, SNC=sinonasal cavity, T=tongue, tongue base & lip	**% Patient samples >70% tumor cells**	**Method of Extraction**
**Original data ID**	**GSE6631**	**GSE2379**	**E-MEXP-44**	**E-MEXP-44_hu6800**	**JCO2010**
# of total samples	44	38	33	35	91
**A**	GSE6631 [61]	22	OC (12)<br>OP (3)<br>OC/OP(1)<br>HP (1)<br>L (4)<br>SNC (1)	100%	Histological
**B**	GSE2379 [62]	31	HP (31)	100%	Histological
**C**	E-MEXP-44 [63]	15	OC (3)<br>OP (5)<br>HP (2)<br>L (5)	100%	Macrodissected
**D**	E-MEXP-44 [63]	12	OC (2)<br>OP (4)<br>HP (2)<br>L (3)<br>Pharynx (1)	100%	Macrodissected
**E**	JCO2010 [47]	63	OC (23)<br>OP (23)<br>HP (12)<br>L (9)<br>T (22)	37%	Haematoxylin and eosin (H&E) stained frozen sections
**F**	GSE686 [48]	60	OC (15)<br>OP (14)<br>HP (7)<br>L (24)	NA	NA
**GSE2837**	GSE2837 [65]	37	OC (18)<br>OP (11)<br>L (8)	100%	H&E stained frozen sections macrodissected
**GSE9844**	GSE9844 [64]	38	T (38)	100%	Laser microdissected
