# Supplementary material for: Single Sample Expression-Anchored Mechanisms Predict Survival in Head and Neck Cancer
Source: PLoS Comput Biol. 2012 Jan 26;8(1):e1002350. doi: 10.1371/journal.pcbi.1002350 (PMC3266878; doi:10.1371/journal.pcbi.1002350)
Supplement: Table S6 — 47 significant Oncogenic FAIME Features of HNSCC confirmed in laser microdissected HNSCC dataset GSE9844. (PDF) [file pcbi.1002350.s013.pdf]

Table S6. 47 significant *Oncogenic FAIME Features of HNSCC* confirmed in laser microdissected HNSCC dataset GSE9844. In dataset (GSE9844) comprising 38 samples of laser microdissected HNSCC tissue and 16 non-tumor tissue control samples from the same anatomical site (tongue squamous), we conducted exactly the same methods as reported in **Figure 2** over datasets A, B and C and identified 751 deregulated FAIME scores out of 2,699 KEGG and GO genesets evaluated. Shown below are the 47 significant GO molecular functions and KEGG pathways in HNSCC tumor samples overlapping with the 57 *Oncogenic FAIME Feature of HNSCC* (q-value<0.05, z-test adjusted for multiple comparisons). For each KEGG pathway or GO molecular function (*Oncogenic FAME Feature of HNSCC*), the FDR and q-value are reported. A positive FAIME fold change corresponds to a FAIME Score increased in HNSCC tissue as compared to the non-tumor control tissue and a negative FAIME fold change corresponds to a FAIME Score decreased in HNSCC tissue as compared to the non-tumor control tissue.

KEGG/GO ID	KEGG pathway/GO function Name	FDR	q-value
hsa05219	Bladder cancer	5.97E-03	4.91E-04
hsa00982	Drug metabolism - cytochrome P450	5.97E-03	4.91E-04
hsa00641	3-Chloroacrylic acid degradation	6.96E-03	5.72E-04
GO:0070330	aromatase activity	1.08E-02	5.95E-04
GO:0003997	acyl-CoA oxidase activity	1.93E-02	1.06E-03
hsa00591	Linoleic acid metabolism	1.53E-02	1.26E-03
hsa00071	Fatty acid metabolism	1.57E-02	1.29E-03
hsa00980	Metabolism of xenobiotics by cytochrome P450	1.57E-02	1.29E-03
hsa05222	Small cell lung cancer	1.57E-02	1.29E-03
hsa00350	Tyrosine metabolism	1.57E-02	1.29E-03
GO:0003995	acyl-CoA dehydrogenase activity	2.63E-02	1.45E-03
GO:0019911	structural constituent of myelin sheath	3.37E-02	1.86E-03
hsa00360	Phenylalanine metabolism	2.74E-02	2.25E-03
hsa04512	ECM-receptor interaction	2.75E-02	2.26E-03
hsa00640	Propanoate metabolism	2.75E-02	2.26E-03
hsa00830	Retinol metabolism	2.75E-02	2.26E-03
GO:0004656	procollagen-proline 4-dioxygenase activity	5.84E-02	3.22E-03
hsa00960	Tropane, piperidine and pyridine alkaloid biosynthesis	4.44E-02	3.65E-03
GO:0004030	aldehyde dehydrogenase [NAD(P)+] activity	7.26E-02	4.01E-03
GO:0050840	extracellular matrix binding	7.26E-02	4.01E-03
hsa00120	Primary bile acid biosynthesis	4.89E-02	4.02E-03
hsa00280	Valine, leucine and isoleucine degradation	5.13E-02	4.21E-03
hsa05200	Pathways in cancer	5.45E-02	4.48E-03
GO:0004029	aldehyde dehydrogenase (NAD) activity	1.06E-01	5.82E-03
GO:0047372	acylglycerol lipase activity	1.11E-01	6.10E-03

hsa00340	Histidine metabolism	7.80E-02	6.41E-03
hsa03030	DNA replication	8.23E-02	6.76E-03
hsa00053	Ascorbate and aldarate metabolism	9.47E-02	7.78E-03
hsa00590	Arachidonic acid metabolism	9.53E-02	7.83E-03
hsa00650	Butanoate metabolism	9.53E-02	7.83E-03
hsa00624	1- and 2-Methylnaphthalene degradation	1.18E-01	9.69E-03
hsa00410	beta-Alanine metabolism	1.18E-01	9.69E-03
hsa00240	Pyrimidine metabolism	1.23E-01	1.01E-02
GO:0048407	platelet-derived growth factor binding	1.87E-01	1.03E-02
hsa00532	Glycosaminoglycan biosynthesis - chondroitin sulfate	1.27E-01	1.04E-02
GO:0003810	protein-glutamine gamma-glutamyltransferase activity	2.09E-01	1.15E-02
GO:0004222	metalloendopeptidase activity	2.17E-01	1.19E-02
GO:0004523	ribonuclease H activity	2.20E-01	1.21E-02
GO:0016538	cyclin-dependent protein kinase regulator activity	2.55E-01	1.40E-02
hsa04110	Cell cycle	1.84E-01	1.52E-02
hsa00561	Glycerolipid metabolism	2.48E-01	2.04E-02
hsa03410	Base excision repair	2.51E-01	2.06E-02
GO:0016705	oxidoreductase activity, acting on paired donors, with incorporation or reduction of molecular oxygen	5.14E-01	2.83E-02
GO:0030247	polysaccharide binding	6.16E-01	3.40E-02
GO:0004301	epoxide hydrolase activity	7.64E-01	4.22E-02
hsa04115	p53 signaling pathway	5.63E-01	4.62E-02
hsa04510	Focal adhesion	5.86E-01	4.81E-02
