# Supplementary material for: Single Sample Expression-Anchored Mechanisms Predict Survival in Head and Neck Cancer
Source: PLoS Comput Biol. 2012 Jan 26;8(1):e1002350. doi: 10.1371/journal.pcbi.1002350 (PMC3266878; doi:10.1371/journal.pcbi.1002350)
Supplement: Table S7 — Non-tumor control samples. (PDF) [file pcbi.1002350.s014.pdf]

Table S7. Non-tumor Control Samples. Shown below are the total number, tissue location, details of epithelial tissue enrichment, and classes of non-tumor controls for each dataset in our study as reported in the original publications cited in the Reference column.

**ID in the manuscript**	**Reference**	**Total # of control samples**	**Tissue location of control sample**	**Enriched for epithelial tissue**	**Classes of control samples** 1. Non-smoker and no history of HNSCC independent control	2. Paired sample from uninvolved, distant site from tumor (>3 cm or contralateral)	3. Paired sample from tumor margin
**A**	GSE6631 [61]	22 (paired)	Mucosae	N	0	100%	0
**B**	GSE2379 [62]	10	Uvula	Y	20%	0	80%
**C**	E-MEXP-44 [63]	15 (paired)	Mucosae	N	0	0	100%
**D**	E-MEXP-44 [63]	12 (paired)	Mucosae	N	0	0	100%
**E**	JCO2010 [47]	14	Mucosae	Y	21%	79%	0
**F**	GSE686 [48]	3	Tonsillar epithelium	Y	100%	0	0
**GSE2837**	GSE2837 [65]	1	Mucosae	Y	NA	NA	NA
**GSE9844**	GSE9844 [64]	16	Tongue Squamous Cells	Y (Microdissected)	NA	NA	NA
