# Supplementary material for: Single Sample Expression-Anchored Mechanisms Predict Survival in Head and Neck Cancer
Source: PLoS Comput Biol. 2012 Jan 26;8(1):e1002350. doi: 10.1371/journal.pcbi.1002350 (PMC3266878; doi:10.1371/journal.pcbi.1002350)
Supplement: Table S8 — Clinical features. (PDF) [file pcbi.1002350.s015.pdf]

Tables S8A-C. Clinical Features. Show below are the percentage of patients with clinical information reported in each dataset from respective publications for TNM stage (**6A**), P53 and HPV status (**6B**), and Smoking and Alcohol history **(6C**).

Table S8A: TNM stage reported for patients in each dataset

Dataset	A	B	C	D	E	F
% of patients with TNM status	100%	100%	NA	NA	100%	73%

Table S8B: P53 and HPV status reported for patient samples in each dataset

Dataset	A	B	C	D	E	F
% of patients with P53 status	100%	NA	NA	NA	NA	NA
% of patients with HPV status	NA	NA	NA	NA	100%	NA

Table S8C: Smoking and Alcohol history reported for patients in each dataset

Dataset	A	B	C	D	E	F
% of patients with smoking history	NA	NA	NA	NA	NA	100%
% of patients with alcohol history	NA	NA	NA	NA	NA	100%
