# Supplementary material for: Single Sample Expression-Anchored Mechanisms Predict Survival in Head and Neck Cancer
Source: PLoS Comput Biol. 2012 Jan 26;8(1):e1002350. doi: 10.1371/journal.pcbi.1002350 (PMC3266878; doi:10.1371/journal.pcbi.1002350)
Supplement: Text S1 — Implementation of Enrichment, GSEA and CORG. (PDF) [file pcbi.1002350.s016.pdf]

Supplementary Text S1: Description of Conventional Enrichment Approaches

The implementation of three conventional enrichment approaches used in this study are described below (hypergeometric enrichment analysis[1,2,3], GSEA[4,5], and CORG: condition-responsive genes[6]). The analyses are applied to three datasets (A-C, **Table 1**) to detect the KEGG and GO-MF terms relevant to HNSCC tumors as compared to normal control tissues.

1. **Enrichment analysis** was selected for comparison because of its wide spread use and success in previous cancer studies. For enrichment analysis of each dataset, the published genes that are differentially expressed between HNSCC tumor group and control normal tissues group of each datasets were used as seeds to an hypergeometric calculator and over-represented GO terms or KEGG pathways were identified at a 5% level of Benjamini and Hochberg adjusted FDR[7]. The identified enriched GO terms were subsequently refined using two methods. For each dataset, conditional hypergeometric tests were run for GO term identification using the Bioconductor package *GOstats* version 2.12.0 and the LMR method[2] (See **Methods**) was applied to remove false positive pvalues inherited in the GO hierarchy.

2. For gene-set analysis (**GSEA**)[5], the Bioconductor package *GSA* was employed using default parameters with the exception of three parameters that were adjusted to obtain a number of predicted GO-MF and KEGG pathways consistent with those obtained with the other enrichment methods. First, paired GSEA tests were used for dataset A and C that contain paired samples, while B was set to the unpaired default value (**Table 1**). Second, to be consistent with other analyses, the minimum number of genes considered for each KEGG/GO term was decreased to 3 genes per geneset (the default of GSEA is 15). Third, the limitation based on a maximum number of mapped genes per term was eliminated (default is 500). For comparison, the resulting raw p-values were adjusted for multiple-testing using the Benjamini and Hochberg method[7] at the default ≤25% FDR for GO molecular function terms as originally published[5]. We are aware that this FDR appears high in comparison to other conventional methods and proceeded in testing FDR≤5% to remain consistent with the hypergeometric test and FAMIE-derived scores, however this FDR was too severe and did not generate as many KEGG pathways or GO terms as the other methods. Thus FDR≤25% is illustrated in **Figure 2**. Normalized values of genes expression of microarrays of each datasets A, B, or C and HNSCC tumor group vs control normal tissue group were used as inputs (**Methods**).

3. The **CORG** (condition-responsive genes[6]) algorithm was applied to normalized values of genes expression of microarrays of each datasets A, B, or C were used as input (**Methods**). As reported by the authors, the Z-transformed score was generated for each gene using expression values across all samples from each dataset, resulting in a mean of zero and a standard deviation of 1 for each gene. Then for each KEGG/GO-MF term, the Z-scores of all member genes were averaged into a combined Z-score representing the activity of a pathway or a molecular function. Subsequently, multiple t-tests across all samples of each dataset were applied to these combined z-scores to compare HNSCC tumors group and control normal tissues group (Bioconductor package *multtest*). The resultant CORG identified features were those KEGG/GO-MF terms with the highest or lowest t-test statistics after greedily searching an "optimal" subset of member genes for each KEGG/GO-MF term[6] (see **Methods and Figure 2** for discussion on CORG thresholds as CORG has not been designed with FDRs).

Reference:

1. Grossmann S, Bauer S, Robinson PN, Vingron M (2006) An Improved Statistic for Detecting Over-Represented Gene Ontology Annotations in Gene Sets. Research in Computational Molecular Biology: Springer Berlin / Heidelberg. pp. 85-98.
2. Lee Y, Yang X, Huang Y, Fan H, Zhang Q, et al. (2010) Network Modeling Identifies Molecular Functions Targeted by miR-204 to Suppress Head and Neck Tumor Metastasis. PLoS Comput Biol 6: e1000730.
3. Falcon S, Gentleman R (2007) Using GOstats to test gene lists for GO term association. Bioinformatics 23: 257-258.
4. Nam D, Kim SY (2008) Gene-set approach for expression pattern analysis. Brief Bioinform 9: 189-197.
5. Subramanian A, Tamayo P, Mootha VK, Mukherjee S, Ebert BL, et al. (2005) Gene set enrichment analysis: a knowledge-based approach for interpreting genome-wide expression profiles. Proc Natl Acad Sci U S A 102: 15545-15550.
6. Lee E, Chuang HY, Kim JW, Ideker T, Lee D (2008) Inferring pathway activity toward precise disease classification. PLoS Comput Biol 4: e1000217.
7. Benjamini Y, Hochberg Y (1995) Controlling the False Discovery Rate: A Practical and Powerful Approach to Multiple Testing. Journal of the Royal Statistical Society Series B (Methodological) 57: 289-300.
