# Supplementary material for: Single Sample Expression-Anchored Mechanisms Predict Survival in Head and Neck Cancer
Source: PLoS Comput Biol. 2012 Jan 26;8(1):e1002350. doi: 10.1371/journal.pcbi.1002350 (PMC3266878; doi:10.1371/journal.pcbi.1002350)
Supplement: Text S2 — R codes to run FAIME. (PDF) [file pcbi.1002350.s017.pdf]

Supplementary Text S2: R codes to run FAIME

```
#################################################################################
# "FAIME" is a new algorithm to predict Functional Analysis of Individual Microarray Expression.
#
# This R code is composed one main function "runFAIME", which is a R wrap for FAIME.         #
# Programmer: Xinan Yang (xyang2 at uchicago.edu)                                            #
# Last updated on Feb 22th, 2010.                                                            #
# Version 2                                                                                  #
#                                                                                            #
# Update recorder:                                                                           #
# Added a parameter weightRank=TRUE; if FALSE, no weighted rank will be used instead         #
#################################################################################

############################
## Pre-required R package        ##
############################
## To install Bioconductor three packages, include the following in your R code.
## source("http://bioconductor.org/biocLite.R")
## biocLite("Biobase")

library("Biobase")

# Declaration of instants which need to be locally modified by user.
ArrayInput                          # mRNA-expression input file name.
Geneset2gene                        # an 2 column matrix mapping between GO to genes
FDR.T = 0.05                        # Threshold to call a gene-set as significant
genewprobe                  # array of microarray probe IDs with annotated gene symbol

##################################################
## The key function to implement FAIME algorithm       ##
##################################################
## parameters (inputs):
## sampleExp: A vector of normalized exp value with probeName
## genemembers:  A vector of probeName of the gene members of a geneset of interest
## weightRank: A logical to decide whether weighted rank to be used, default is TRUE.
##        if weithRank="mild", the exp( (rank-N)/N) was applied which controls the weights
## between 0 and 1
## output:
## y: A score assigned to each microRNA

        FAIME <- function(sampleExp, genemembers, na.last=TRUE, weightRank,
logCheck=FALSE)
{
        # check if it log transformed, if not, log transformed #
        if (logCheck) {       # by this transformation, negative value will be scaled to NA #
                        if (max(sampleExp) > 20) sampleExp <- log2(SampleExp)
                       }

  if (any(is.na(names(sampleExp))))  stop("Please input sampleExp with probe IDs")
        allGenes <- names(sampleExp)
        N <- length(allGenes)
        nongenemembers <- allGenes[-which(allGenes %in% genemembers)]
```

```
	# Step 1: Calculation of weighted rank of gene expression #
	# Rank from the lowest to highest, thus the leading up-regulated genes get the higher 
weighted score #
	rankedExp <- rank(sampleExp, na.last=na.last)
	if(weightRank=="mild")  {
					rankscore <- rankedExp*exp((rankedExp-N)/N)
					  } else {
						if (weightRank) {
						rankscore <- rankedExp*exp(rankedExp/N)
								} else {
									rankscore <- rankedExp
									}
						}


	# Step 2: Calculate F-score for each individual gene-set per a sample using mRNA 
expression of their gene members and that of its none-members #
	ST <- sum(rankscore[genemembers])/length(genemembers)
	SN <- sum(rankscore[nongenemembers])/length(nongenemembers)
	y <- ST - SN
	return(y)
}


#################################
## The main function to run FAIME ##
#################################
## parameters (inputs):
## dat: An expression matrix, row for a probeName and column for a sample
## genewprobe: A vector of gene Symbol for rows of dat, the names of which is probeName
## geneset2gene: An one-to-one mapping matrix with two columns,
##	the 1st column is geneset ID/name, and the 2nd is its gene members
## weightRank: A logical to decide whether weighted rank to be used, default is TRUE.
## if weithRank="mild"  the exp( (rank-N)/N) was applied which controls the weights
##	between 0 and 1
## output:
## res: A matrix of transformed microRNA profiling, the score calculated by FAIME

runFAIME <- function(dat, genewprobe, geneset2gene, na.last=TRUE, weightRank=TRUE)
{
	if  (class(dat)=="ExpressionSet")  dat <- exprs(dat)
	if  (is.null(names(genewprobe))) stop("Please input the zz as a named vector for gene 
Symbol!")
	allSym <- genewprobe [rownames(dat)]
	seeds <- unique(geneset2gene[,1])
	res <- matrix(nrow=length(seeds), ncol=ncol(dat))
	rownames(res) <- seeds
	colnames(res) <- colnames(dat)
	geneIDs <- rownames(dat)

	for(i in 1:length(seeds))
	{
		genemembers <- geneset2gene[which(geneset2gene [,1]==seeds[i]),2]
```

```
		targetP <- unlist(allSym[which(allSym %in% genemembers)] )
		for (j in 1:ncol(dat))
		{
			oneSampleExp <- dat[,j]
			names(oneSampleExp) <- geneIDs
			res[i,j] <- FAIME(oneSampleExp,  genemembers=names(targetP),
						na.last=na.last, weightRank=weightRank)
		}
	}
	length(which(is.na(res[,1])))  # there might be geneset without measured member gene on this array #
	return(res)
}
```
